# Identifying critical windows of air pollution exposure during preconception and gestational period on birthweight: a prospective cohort study

**DOI:** 10.1186/s12940-023-01022-6

**Published:** 2023-10-19

**Authors:** Jiawen Liao, Yi Zhang, Zhenchun Yang, Chenyu Qiu, Wu Chen, Junfeng Jim Zhang, Kiros Berhane, Zhipeng Bai, Bin Han, Jia Xu, Yong-hui Jiang, Frank Gilliland, Weili Yan, Guoying Huang, Zhanghua Chen

**Affiliations:** 1 Department of Population and Public Health Sciences, Keck School of Medicine, University of Southern California, Los Angeles, CA United States of America; 2 Department of Clinical Epidemiology & Clinical Trial Unit, Children’s Hospital of Fudan University, National Children’s Medical Center & Shanghai Key Laboratory of Birth Defects, Shanghai, China; 3 Duke Global Health Institute, Durham, NC United States of America; 4 Division of Environmental Science and Policy, Nicholas School of the Environment, Duke University, Durham, NC United States of America; 5 Department of Biostatistics, Mailman School of Public Health, Columbia University, New York, NY United States of America; 6 State Key Laboratory of Environmental Criteria and Risk Assessment, Chinese Research Academy of Environmental Sciences, Beijing, China; 7 Department of Genetics, Neuroscience, and Pediatrics, Yale University School of Medicine, New Haven, CT United States of America

**Keywords:** Air Pollutant, Small for gestational age, Large for gestational age, Preconception, Pregnancy, Birthweight

## Abstract

**Background:**

Few studies have assessed air pollution exposure association with birthweight during both preconception and gestational periods.

**Methods:**

Leveraging a preconception cohort consisting of 14220 pregnant women and newborn children in Shanghai, China during 2016–2018, we aim to assess associations of NO_2_ and PM_2.5_ exposure, derived from high-resolution spatial-temporal models, during preconception and gestational periods with outcomes including term birthweight, birthweight Z-score, small-for-gestational age (SGA) and large-for-gestational age (LGA). Linear and logistic regressions were used to estimate 3-month preconception and trimester-averaged air pollution exposure associations; and distributed lag models (DLM) were used to identify critical exposure windows at the weekly resolution from preconception to delivery. Two-pollutant models and children’s sex-specific associations were explored.

**Results:**

After controlling for covariates, one standard deviation (SD) (11.5 μg/m^3^, equivalent to 6.1 ppb) increase in NO_2_ exposure during the second and the third trimester was associated with 13% (95% confidence interval: 2 – 26%) and 14% (95% CI: 1 – 29%) increase in SGA, respectively; and one SD (9.6 μg/m^3^) increase in PM_2.5_ exposure during the third trimester was associated with 15% (95% CI: 1 – 31%) increase in SGA. No association have been found for outcomes of birthweight, birthweight Z-score and LGA. DLM found that gestational weeks 22–32 were a critical window, when NO_2_ exposure had strongest associations with SGA. The associations of air pollution exposure tended to be stronger in female newborns than in male newborns. However, no significant associations of air pollution exposure during preconception period on birthweight outcomes were found.

**Conclusion:**

Consistent with previous studies, we found that air pollution exposure during mid-to-late pregnancy was associated with adverse birthweight outcomes.

**Supplementary Information:**

The online version contains supplementary material available at 10.1186/s12940-023-01022-6.

## Introduction

Ambient air pollution, especially its components of particulate matter with equal to or less than 2.5 μm aerodynamic diameter (PM_2.5_) and nitrogen dioxide (NO_2_), is one of most important environmental health risk factors [1, 2]. Even though annual averages of ambient air pollution levels have generally declined in Shanghai, a mega city in China, over the past 5 years [3, 4], PM_2.5_ and NO_2_ are still well above World Health Organization (WHO) ambient air quality guideline of PM_2.5_ as 5 μg/m^3^ and NO_2_ as 10 μg/m^3^ [5]. Over 1 million premature deaths due to lung cancer, cardiovascular diseases and lower-respiratory infection and chronic lung disease can be attributed to excessive levels of air pollution in China [1, 2]. In addition to asserting excess mortality, exposure to high levels of air pollution during pregnancy has also been associated with adverse birth outcomes, including preterm birth, low birthweight (LBW), small for gestational age (SGA), large for gestational age (LGA) and birth defects [6–13]. Infants born higher- or lower-than normal birthweight have been shown to be associated with abnormal growth trajectory and linked to increased risk of childhood obesity and other cardiometabolic disorders later in life [14–16].

While existing evidence suggests a link between PM_2.5_ and NO_2_ exposure during gestational period and birthweight [7, 17–19], the critical exposure time windows in the pregnancy period were inconsistent [12, 20]. On the other hand, the evidence is extremely limited concerning pre-conception period air pollution exposure and birth outcomes. Only a few studies have assessed the associations of air pollution during preconception period on birthweight [19, 21–24]. These studies assessed the associations of exposures to several air pollutants during the 3-month preconception period with the risk of adverse birthweight outcomes such as SGA. However, no consistent associations have been found for preconception exposure to PM_2.5_ and NO_2_ air pollution [21–24]. Preconception is a critical developmental window for gametogenesis; and air pollution exposure from both mothers and fathers during preconception or early stages of pregnancy could be as important as during pregnancy to health of children in the future [25]. Previous studies have found that air pollution exposure during these critical gametogenesis period could lead to adverse effects on development of sperm and ova cells [26, 27], disrupting first cell lineage segregation at the blastocyst stage [28], diminishing ovarian reserve [29], and finally leading to long-term adverse outcomes of fetal and neonatal development [30, 31]. Considering the potential importance of preconception exposure in fetal body growth, we leveraged the rich data resource of the Shanghai Pre-conception Cohort (SPCC) to investigate the effect estimates of air pollution during both preconception and gestational period on birthweight outcomes. We aim to identify critical time windows of exposure associated with birth outcomes during both the preconception period and the gestational period. In addition, we also aim to assess whether these associations differed by children’s sex, in order to understand sex-specific relationships between prenatal air pollution exposure and birthweight outcomes.

## Method

### Study design and population

This Growth and Air Pollution in Preconception (GAAP) study was based on pregnancy-planning women and men enrolled in the Shanghai Preconception Cohort (SPCC). The SPCC was established with its primary aim to investigate the associations of parental periconceptional nutritional factors with congenital heart disease, child growth and development and pediatric diseases. More details of SPCC cohort were reported elsewhere [32]. Briefly, SPCC enrolled N = 34,759 women at preconception or antenatal care clinics from 28 maternity institutions in 10 districts of Shanghai, between March 2016 to December 2018. Participants of the SPCC cohort who delivered between March 2016 to December 2018 and registered residential address during peri-conception were included in the GAAP study. The exclusion criteria of the GAAP study included (1) participants missing residential address or (2) moving out of Shanghai city. The GAAP study protocols were approved by the Ethics Committee of the Children’s Hospital of Fudan University (IRB number: 201,649), Duke University (IRB number: 00000560) and University of Southern California (IRB number: HS-19-00306), respectively.

### Participants follow up and covariates

Participating women completed the baseline questionnaire of key demographics following their pregnancy consultation visits to a clinic (i.e., the preconception visits), after they signed a consent to participate. Information collected included maternal age, ethnicity, education levels and employment. When participants visited the first antenatal care around 14-weeks of gestational age, another questionnaire was administered to collect lifestyle covariates, including maternal smoking, exposure to secondhand smoking, nutrition supplementation information during the periconception period such as folic acid supplement. We defined maternal smoking and secondhand smoke as whether women participants self-reported active smoking and exposed to cigarette smoke from family members or colleagues in working space between 3-month preconception and first trimester, respectively. Routine antenatal care electronic medical record data were obtained from the Maternal Clinic Antenatal Medical Record System (MCAMRS), managed by the Shanghai Center for Women and Children’s Health, including gestational weeks measured by ultrasound or last menstrual period, anthropometry measurements of women participants such as height, gestational weight, last menstrual period, and childbearing history collected at first antenatal care visit. Maternal body mass index (BMI) at early pregnancy was calculated using maternal weight and height and categorized as normal (BMI < 24 kg/m^2^) or overweight (BMI > = 24 kg/m^2^ and BMI < 28 kg/m^2^) or obesity (BMI > = 28 kg/m^2^) according to the definition of the Chinese population recommend by Working Group on Obesity in China [33]. Participants were followed until delivery. Newborn information was also extracted from MCAMRS, including delivery date, delivery mode, birthweight, child’s sex, birth defects and pregnancy complications if any. Based on the conception date and delivery date, we calculated the gestational age in week at delivery for each newborn. We only included children born full-term [gestational age > = 37 weeks, N = 14,220 (95.0%)] in the analysis. The first trimester was defined as the period during gestational week 0–13 (day 0 – day 91), the second trimester was gestational week 14–26 (day 92 – day 182); and the third trimester was from gestational week 27 (day 183) to delivery. Additionally, the season of delivery (spring/summer/fall/winter), weekly average temperature (°C) and relative humidity in Shanghai were derived from the data retrieved from National Centers for Environmental Information of United States (https://www.ncdc.noaa.gov/).

### Preconception air pollution exposure

Air pollution exposures were assessed based on a spatial-temporal model of PM_2.5_ and NO_2_ at three-day temporal resolution predicted at each participant’s residential address extracted from MCAMRS, between October 2012 and December 2019 [34], which covers entire preconception and pregnancy period of cohort of this study. This spatial-temporal model was specifically developed for this study based on smoothed temporal trends and the land-use regression approach in a universal kriging structure [35]. This model has shown good performance at both short-term (three-day average) and long-term (7-year average) scales, with leave-one-out cross-validation (LOOCV) rooted-mean square error (RMSE) 2.01 μg/m^3^ (R^2^ = 0.72) for PM_2.5_ and 3.07 μg/m^3^ (R^2^ = 0.87) for NO_2_ in the long-term averages and 5.82 μg/m^3^ (R^2^ = 0.94) for PM_2.5_ and 7.50 μg/m^3^ (R^2^ = 0.83) for NO_2_ in three-day averages [34]. We used this model to predict three-day average concentrations of PM_2.5_ and NO_2_ at each participant’s residential address. Then, based on the conception date and delivery date, we calculated weekly air pollution exposure during pregnancy as gestational air pollution exposure for each 7-day period from conception date, and estimated trimester-specific exposures to PM_2.5_ and NO_2_. Additionally, we estimated weekly exposures from conception date up to 15 weeks before conception date, and we used the mean of the air pollution exposure levels from conception to 12 weeks before conception date as the average preconception exposure.

### Birthweight outcomes

Birth weights in gram of newborns were extracted from the MCAMRS, which recorded birthweight of every newborn in Shanghai and matched with study participants’ information. Birthweight was measured to nearest gram and recorded into the system within 72 h after birth by a nurse in obstetrics. We estimated sex- and gestational age-specific birthweight Z-scores and percentiles of each newborn using recorded birthweight, gestational age at delivery and reference percentile chart for Chinese representative children [36]. Based on the 10th and 90th percentile cut-offs, we categorized the sex and gestational age-specific birth weight into small for gestational age (SGA) as less than 10th percentile, appropriate for gestational age (AGA) as between 10th and 90th percentile, and large for gestational age (LGA) as above 90th percentile. The outcome variables in this study included birth weight in gram, birthweight Z scores, and gestational age-specific birth weight categories (SGA and LGA).

### Statistical analysis

Firstly, we assessed the 12-week preconception and trimester-averaged gestational period air pollution associations with birth weight outcomes. We applied multivariate regressions with linear and non-linear exposure-response functions to assess effect estimates of air pollution during these periods on birthweight and birthweight Z-score. We applied logistic regressions to assess the associations with SGA and LGA. We included pre-specified covariates to adjust in the models based on previous studies and potential confounding effects of covariates [21–23, 37]. For multivariate regression models of birthweight, we included covariates including maternal age, ethnicity, education, occupation, maternal BMI at first trimester, maternal gravidity, maternal smoking status, exposure to secondhand smoking, season of delivery, gestational age, children’s sex, temperature, and relative humidity. For outcomes of birthweight Z-score, SGA and LGA, since gestational age and children’s sex were incorporated for the calculations, we included all the covariates other than gestational age and child’s sex. 

Secondly, we attempted to identify the critical time window for birth weight outcomes during the preconception and pregnancy period and non-linear exposure-response associations of maternal air pollution on birthweight outcomes. In doing so, we applied distributed lag non-linear models (DLNM) to evaluate weekly-specific time-varying effect estimates of maternal exposure to air pollution [38]. We built DLNM models separately for each pollutant of PM_2.5_, and NO_2_. The framework of DLNM was based on a “cross-basis” function to flexibly model both exposure-response associations and lag structure of the associations between exposures at different times. DLNM included weekly air pollution data simultaneously and estimated associations between birthweight outcomes and air pollutant exposure for a given week after controlling for exposure at all other weeks, based on an assumption that association varies smoothly as a function of week. We included the weekly air pollution 15 weeks before conception as the pre-conception period exposure, and weekly air pollution exposure for 37 weeks after conception as the gestational period exposure. Only children born full-term ( > = 37 weeks) were included in the analysis, and for children born with more than 37 weeks, only air pollution exposure for first 37 weeks of gestation was included in the analysis. To develop DLNM model, we firstly investigated the non-linearity of exposure-response effect estimates of air pollution on the outcomes, and we found linear exposure-response provided best fit for minimizing Akaike Information Criteria (AIC). Supplemental Information (SI) Figure S1 shows the difference exposure-response function curves and associated AIC, comparing linear, quadratic polynomial and cubic polynomial functions. Therefore, we used simplified distributed-lag linear model (DLM) in the analysis. Then, we modeled the exposure lag-response function of weekly air pollution in DLM. We used the natural splines [39, 40] to model this smooth lag function based on minimizing AIC with degree of freedom (*df*) range from 3 to 7, with knots placed at equal space at all lags. Based on the AIC values, the natural cubic spline of the lag function with 4 *df* provided best fit of PM_2.5_ on birthweight and birthweight Z-score, SGA and LGA and best fit for NO_2_ on SGA and LGA; *df* of 5 provided best fit for NO_2_ on birthweight Z-score and birthweight. Like in the trimester-averaged air pollution models, same covariates were included in the DLM analysis.

Following these analyses, we conducted stratified analysis by children’s sex, and assessed whether associations of air pollution on birthweight differed by children’s sex. Lastly, we conducted sensitivity analysis to confirm the robustness of the findings. In the first sensitivity analysis, we assessed the weekly air pollution associations with the birthweight outcomes separately. In the second sensitivity analysis, we conducted two-pollution air pollution model, included both NO_2_ and PM_2.5_ trimester-averaged air pollution exposure in the model. Lastly, we conduct sensitivity analysis on birthweight without adjusting gestational age at birth, since gestational age could be an intermediate factor between air pollution exposure and birthweight outcomes. R (version 4.1.3) with *mgcv* and *dlnm* packages was used in the statistical analyses.

## Results

### Population characteristics

**Fig. 1:**
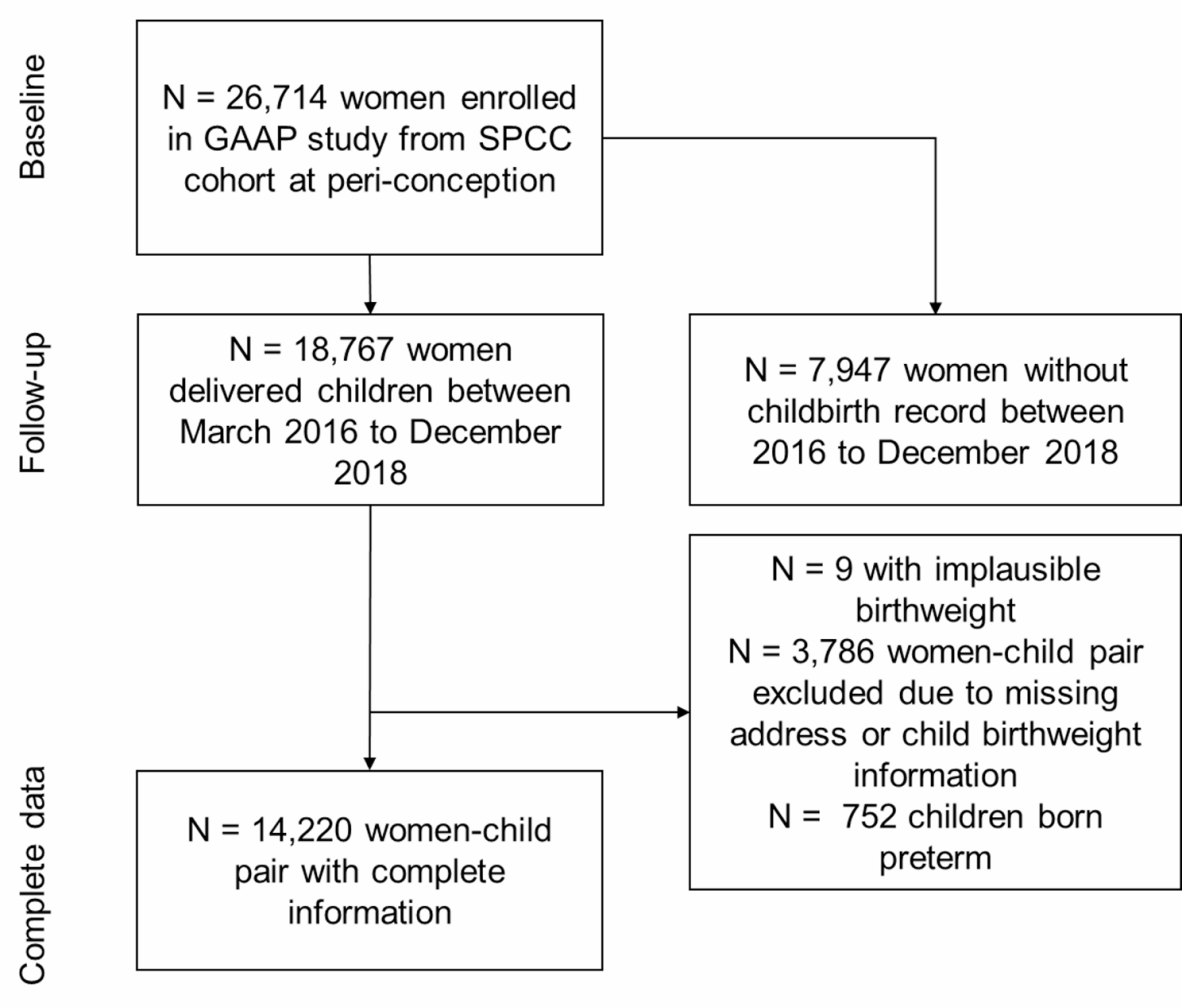
Flow diagram of the Growth and Air Pollution in Preconception (GAAP) Cohort participant screening, showing the final sample size of 14,220 used in the main data analysis

Between March 2016 to December 2018, 18767 pregnant women, from the total of 34759 SPCC cohort participants (26714 women participants), delivered a baby and were potentially eligible for the GAAP study. Among them, 18,767 participants had complete information on newborns’ birthweight and sex as well as gestational age at birth. We excluded 9 children with birthweight less than 200 gram (an implausible value) and removed 3786 additional mother-child pairs due to missing baseline address information, air pollution exposure assessment and outcome health information, and 752 children who were born with gestational age less than 37 weeks (preterm birth). We excluded preterm birth infants mainly due to that could be associated with other maternal factors such as complications, maternal age and delivery mode in China [41], potentially inducing bias in the analysis. Consequently, 14220 mother-child pairs had complete information and were included in our main analysis in this study. Figure 1 illustrates the flow diagram of the cohort of this study. In Table 1, we show the participants’ characteristics at enrollment during preconception or early pregnancy period. This shows that the mean (SD) of the maternal age is 33.4 (3.8) years at conception date. Near half of the participants did not report their ethnicity (N = 7206, 50.7%), while the rest of the women participants were of Han ethnicity (N = 6885, 48.4%). Additionally, participants mostly reported having a college degree (N = 9703, 68.2%), and working as a manufactural worker, office clerk or self-employed (N = 8257, 58.1%), followed by manager, entrepreneur, or technician (N = 4834, 34.0%). A very small proportion of the women reported smoking during preconception and early pregnancy (N = 155, 1.1%), whereas more women (N = 2318, 16.3%) reported that they were exposed to second-hand smoke during this period. Most women had no previous baby delivery (N = 10962, 77.2%), while 3,199 (22.4%) reported they had one delivery before this pregnancy. The mean (SD) maternal body mass index (BMI) in the first trimester was 20.7 (4.4) kg/m^2^. The vast majority (72.6%) of the women had BMI in the healthy range (18.5 ‒ 24 kg/m^2^), while 11.2% in the overweight range of 24–28 kg/m^2^ and 2.7% in the obese category of ≥ 28 kg/m^2^.

### Birthweight outcomes

In Table 1, we also summarize birthweight data for full-term birth children. The mean (SD) of the birthweight was 3358.7 (402.0) gram, and the Z-score of the birthweight was 0.2 (1.0) based on the Chinese infant birthweight chart [36]. Among all the full-term birth children included in our analysis, 820 (5.8%) and 1819 (12.8%) had birthweight classified as small for gestational age (SGA) and large for gestational age (LGA). The mean (SD) of the gestational age of these births was 39.2 (1.1) weeks, and slightly more than half of the births were delivered as vaginal birth (N = 8650, 60.8%). Deliveries occurred mostly in the fall season (N = 4189, 29.5%), followed by in summer (N = 3514, 24.7%), spring (N = 3253, 22.9%) and winter (N = 3264, 23.0%). Slightly more than half (N = 8070, 56.8%) of the children delivered were boys.

**Table 1:** Summary of Study Participants’ Characteristics and Birthweight Outcome and Meteorology Variables (N = 14220)

Characteristics	Mean (SD) or n (%)	
**Birthweight Outcomes**		
Birthweight (g), mean (SD)	3358.7	(402.0)
Z-score, mean (SD)	0.2	(1.0)
Small for gestational age (SGA), n (%)	820	(5.8%)
Large for gestational age (LGA), n (%)	1819	(12.8%)
**Maternal variables**		
Maternal age in years during first antenatal visit	33.4	(3.8)
Age < = 25	111	(0.8%)
25 < Age < = 30	2823	(19.9%)
30 < Age < = 35	7646	(53.8%)
35 < Age	3640	(25.5%)
Maternal ethnicity, n (%)		
Han	6885	(48.4%)
Other ethnic minority	129	(0.9%)
Not reported	7206	(50.7%)
Education level, n (%)		
High school or less	1155	(8.1%)
College/undergraduate level	9703	(68.2%)
Above college	2344	(16.5%)
Not reported	1017	(7.2%)
Number of deliveries before, n (%)		
Zero	10,962	(77.1%)
One	3199	(22.5%)
Two or above	59	(0.4%)
Maternal first trimester BMI, mean (SD)	20.7	(4.4)
Maternal first trimester BMI category, n (%)		
BMI < 18.5 kg/m^2^	1921	(13.5%)
18.5 kg/m^2^ <=BMI < 24 kg/m^2^	10,360	(72.9%)
24 < = BMI < 28 kg/m^2^	1577	(11.1%)
BMI > = 28 kg/m^2^	362	(2.5%)
Active smoking between preconception and early pregnancy, n (%)		
Yes	155	(1.1%)
No	13,085	(92.0%)
Missing	980	(6.9%)
Environmental smoking between preconception and early pregnancy, n (%)		
Yes	2318	(16.3%)
No	10,911	(76.7%)
Missing	991	(7.0%)
Occupation, n (%)		
Worker/ office clerk / Self-employed	8257	(58.1%)
Manager/Technician/Entrepreneur	4834	(34.0%)
Missing/unemployed	1129	(7.9%)
Folic acid supplementation during preconception and early pregnancy		
Yes	9963	(70.1%)
No	4257	(29.9%)
**Children variable**		
Birth season, n (%)		
Spring (March ‒ May)	3253	(22.9%)
Summer (June ‒ August)	3514	(24.7%)
Fall (September ‒ November)	4189	(29.5%)
Winter (December ‒ February)	3264	(23.0%)
Delivery mode		
Vaginal birth	8650	(60.8%)
Caesarean section	5570	(39.2%)
Birth term/gestational age (weeks), mean (SD)	39.2	(1.1)
Children’s sex		
Male	8070	(56.8%)
Female	6150	(43.2%)

SGA is defined as birthweight Z-score is below 10th percentile, and LGA is defined as above 90th percentile based on [36]

### Air pollution exposure assessment

In Table 2, we show the levels of NO_2_ and PM_2.5_ predicted for the 3-month preconception period, first trimester, second trimester, and third trimester averages, respectively. The correlations among pollutant exposures during the four periods are listed in Figure S2, showing that at the same period, NO_2_ and PM_2.5_ had a relatively high correlation (spearman correlation ρ > 0.75), while crossing different periods, the correlations were relatively low for NO_2_ (spearman correlation − 0.5 < ρ < 0.5) and the correlations were moderate and negative for PM_2.5_. Between preconception and second trimester and between the first and third trimester PM_2.5_ exposures had correlation coefficient of -0.72 and − 0.76, respectively. The density plots of air pollution exposure levels during these periods of time are shown in Figure S3. Approximately 66% of the NO_2_ exposure levels during pre-conception and gestational periods were above annual National Ambient Air Quality Standard in China of 40 μg/m^3^, and 85% of the PM_2.5_ exposure levels were above annual National Ambient Air Quality Standard in China of 35 μg/m^3^ [42].

**Table 2:** Summary of Ambient Air Pollution Exposure Levels During 3-month Preconception period, first trimester, second trimester, third trimester of pregnancy and the entire pregnancy period, unit: μg/m^3^

Time point	NO_2_		PM_2.5_	
	Mean (SD)	Median (Q1 – Q3)	Mean	Median (Q1 – Q3)
Preconception	42.1 (9.9)	41.6 (34.1, 49.5)	38.3 (8.5)	37.9 (30.6, 45.0)
First Trimester	42.9 (10.8)	43.0 (34.5, 51.1)	38.9 (9.3)	38.9 (30.9, 46.8)
Second Trimester	42.0 (10.9)	41.3 (33.6, 50.2)	37.6 (9.0)	36.9 (30.3, 44.8)
Third Trimester	41.5 (12.5)	39.8 (31.3, 50.2)	36.2 (10.4)	35.4 (27.4, 44.2)
Whole pregnancy	42.8 (11.5)	41.4 (33.1, 50.5)	37.5 (9.6)	37.6 (29.5, 45.5)

Unit of PM_2.5_ and NO_2_ air pollution exposure: μg/m^3^

### Effect estimates of preconception and gestational air pollution exposure on birth weight outcomes

Table 3 shows the results of multivariate regression analysis of single pollutant NO_2_ and PM_2.5_ effect estimates on birthweight, birthweight Z-score, and logistic regression of small for gestational age (SGA) and large for gestational age (LGA). Trimester-specific exposures showed strongest and most consistent associations with small-for-gestational (SGA), whereas their associations with birthweight and birthweight Z-score, and large-for-gestational age were non-significant. Specifically, we found that a standard deviation (11.5 μg/m^3^, equivalent to 6.1 ppb) increase in NO_2_ exposure was associated with the increased odds ratio (OR) of small for gestational age (SGA) during the second (OR: 1.13, 95% confidence interval (CI): 1.02 – 1.26) and third trimester (OR: 1.14, 95% CI: 1.01 – 1.29) after controlling for covariates. We also found a significant positive association between PM_2.5_ and SGA during the third trimester (OR: 1.15, 95%: 1.01 – 1.31) per 1 standard deviation (9.6 μg/m^3^) increase.

**Table 3:** Differences in birthweight (g), Z-score and odds ratios (ORs) of SGA and LGA per 1 standard deviation (SD) increase in preconception and trimester-averaged NO_2_ and PM_2.5_ exposure (9.6 μg/m^3^ for PM_2.5_ and 11.5 for NO_2_), respectively. For birthweight outcome, models are controlled for maternal age, ethnicity, education, occupation, maternal BMI at first trimester, maternal gravidity, maternal smoking status, exposure to secondhand smoking, season of delivery, gestational age, children’s sex, temperature, and relative humidity; for Z-score, SGA and LGA outcomes, models controlled for same covariates except for children’s sex and gestational age

		NO_2_				PM_2.5_			
Outcome	Time period	Point Estimate	P value	LCI	UCI	Point Estimate	P value	LCI	UCI
Birthweight	Preconception	0.17	0.97	-9.59	9.92	8.72	0.18	-4.13	21.56
	1st Trimester	-6.32	0.21	-16.15	3.50	-4.63	0.42	-15.76	6.50
	2nd Trimester	-6.88	0.18	-16.96	3.21	-3.85	0.52	-15.45	7.76
	3rd Trimester	-0.09	0.99	-11.44	11.26	-0.61	0.92	-12.17	10.96
	Whole pregnancy	-4.12	0.318	-12.202	3.961	-4.00	0.40	-13.31	5.32
Z-score	Preconception	0.001	0.927	-0.024	0.026	0.019	0.268	-0.014	0.052
	1st Trimester	-0.014	0.276	-0.039	0.011	-0.011	0.471	-0.039	0.018
	2nd Trimester	-0.009	0.503	-0.035	0.017	0.003	0.854	-0.027	0.033
	3rd Trimester	0.003	0.847	-0.026	0.032	0.004	0.792	-0.026	0.034
	Whole pregnancy	-0.006	0.570	-0.027	0.015	0.000	0.999	-0.024	0.024
		OR	P value	LCI OR	UCI OR	OR	P value	LCI OR	UCI OR
SGA	Preconception	1.02	0.69	0.92	1.14	0.96	0.62	0.84	1.11
	1st Trimester	0.98	0.66	0.88	1.08	0.93	0.24	0.82	1.05
	2nd Trimester	**1.13**	**0.03**	**1.02**	**1.26**	1.08	0.22	0.95	1.23
	3rd Trimester	**1.14**	**0.04**	**1.01**	**1.29**	**1.15**	**0.04**	**1.01**	**1.31**
	Whole pregnancy	1.04	0.29	0.97	1.12	1.05	0.27	0.96	1.15
LGA	Preconception	0.99	0.86	0.92	1.07	1.02	0.66	0.92	1.13
	1st Trimester	0.99	0.74	0.92	1.06	0.95	0.23	0.87	1.03
	2nd Trimester	1.04	0.28	0.97	1.13	1.05	0.35	0.95	1.15
	3rd Trimester	1.04	0.38	0.95	1.14	1.05	0.28	0.96	1.15
	Whole pregnancy	1.02	0.43	0.97	1.08	1.01	0.77	0.95	1.08

**Fig. 2:**
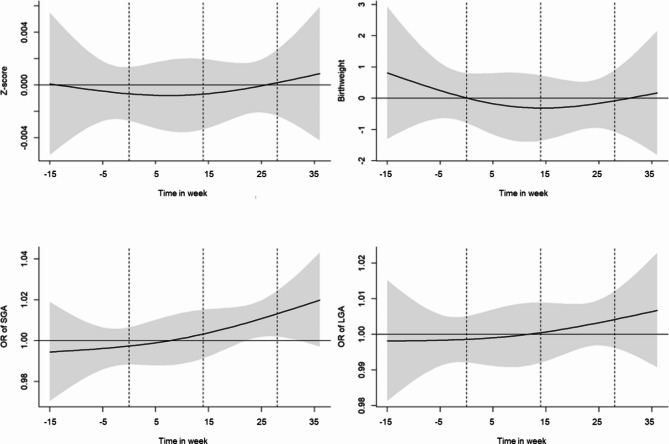
Differences in birthweight Z-score, birthweight, SGA and LGA associated with 1 IQR increase (17.4 μg/m^3^, equivalent to 9.26 ppb) of NO_2_ during preconception and gestational periods. Distributed lag models were adjusted for the maternal age, ethnicity, education, occupation, maternal BMI at first trimester, maternal gravidity, maternal smoking status, exposure to secondhand smoking, season of delivery, gestational age (only for birthweight), children’s sex (only for birthweight), temperature, and relative humidity. The x-axis indicated the time in week of gestation (i.e. conception date is 0 week). The dashed line separated preconception period, first trimester, second trimester and third trimester from left to right, respectively

**Fig. 3:**
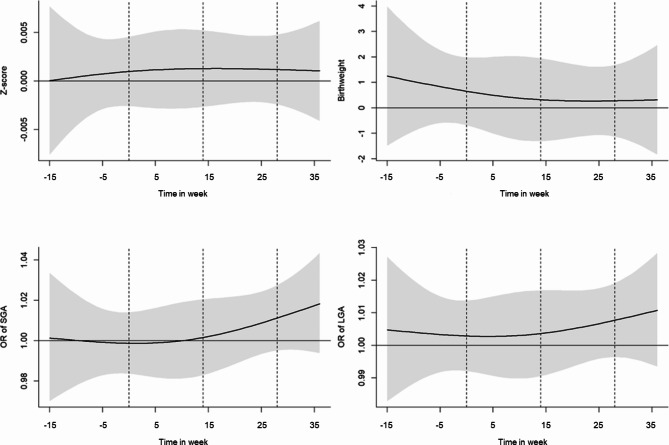
Differences in birthweight Z-score, birthweight, SGA and LGA associated with 1 IQR (16 μg/m^3^) increase of PM_2.5_ concentrations during preconception and gestational periods. Distributed lag models were adjusted for the maternal age, ethnicity, education, occupation, maternal BMI at first trimester, maternal gravidity, maternal smoking status, exposure to secondhand smoking, season of delivery, gestational age (only for birthweight), children’s sex (only for birthweight), temperature, and relative humidity. The x-axis indicated the time in week of gestation (i.e. conception date is 0 week). The dashed line separated preconception period, first trimester, second trimester and third trimester from left to right, respectively

Figure 2 illustrates the associations of 1 interquartile range (IQR, 17.4 μg/m^3^, equivalent to 9.3 ppb) increase in NO_2_ exposure on birthweight, birthweight Z-score, SGA and LGA, showing the lag-response relationships for weekly NO_2_ exposures during the preconception and gestational periods. Figure 3 illustrates the associations of the 1 IQR (16 μg/m^3^) increase in weekly PM_2.5_ exposures on birthweight, birthweight Z-score, SGA and LGA. Similar to the results of trimester-averaged air pollution exposure models, we found significant positive associations between NO_2_ and SGA at later pregnancy. We found that increased weekly NO_2_ exposure from 1st quartile (33.1 μg/m^3^) to 3rd quartile (50.5 μg/m^3^) during the 22nd gestational week to the 32nd gestational week was associated with increased odds of SGA (for example, OR = 1.02, 95% CI: 1.00–1.04 in gestational week 28) (SI Table S1 for numerical results for all week). However, we did not see significant associations between weekly PM_2.5_ exposures with birthweight Z-score, birthweight, SGA, or LGA during the preconception and gestational periods (Fig. 3 and SI Table S2).

**Fig. 4:**
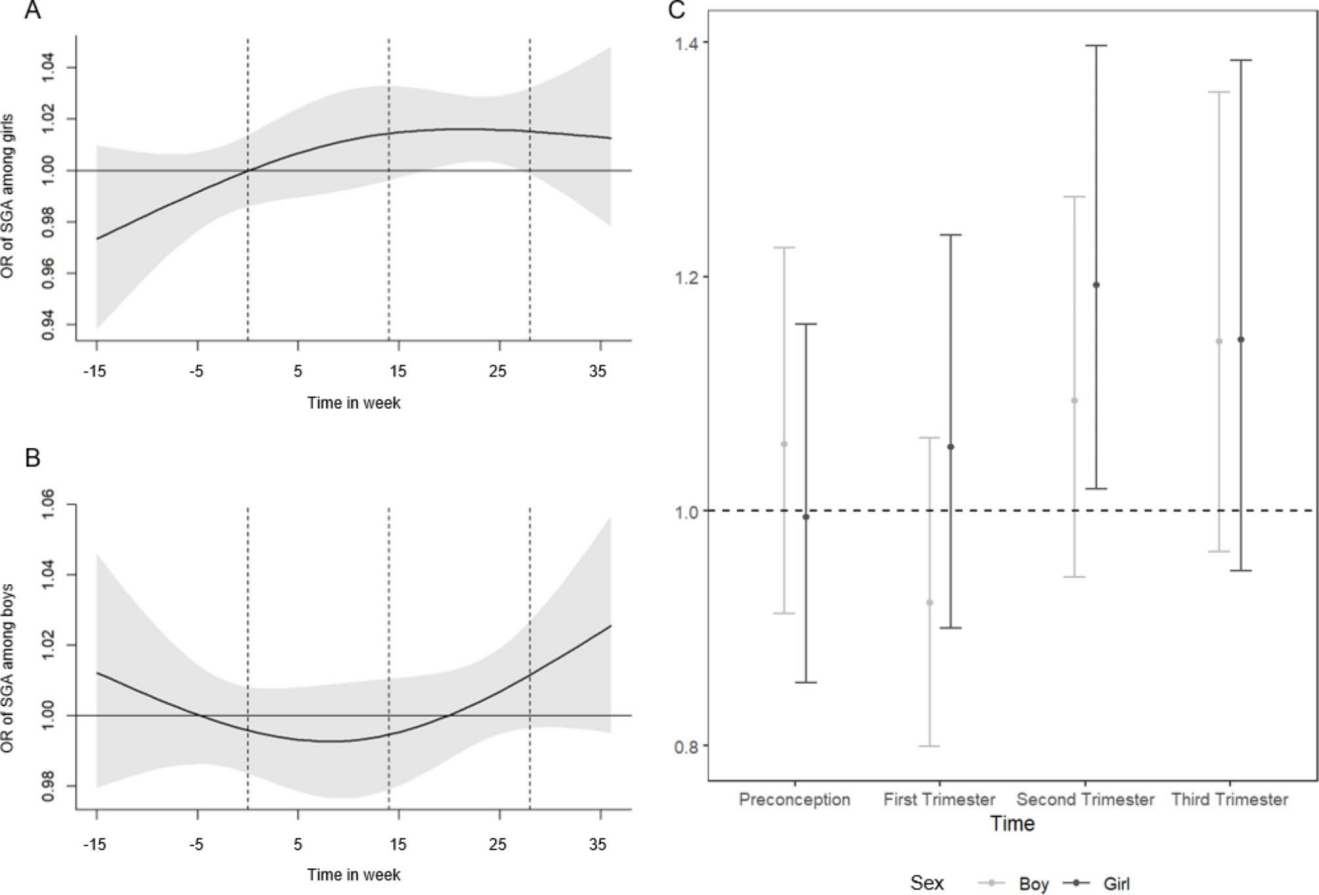
Sex-stratified analysis of NO_2_ exposure during 3-month preconception and gestational period on Odds Ratio (OR) of SGA. **A**: DLM of NO_2_ on SGA using DLM among female children (girl); **B**: DLM of NO_2_ on SGA using DLM among male children (boy); **C**: preconception and trimester-averaged model by newborns’ sex. Same covariates were adjusted as full models

In the sex stratified analysis, we found that girls were more vulnerable to air pollution exposure than boys during later pregnancy, as NO_2_ exposure at later pregnancy was significantly associated with increased odds of SGA in girls but not in boys shown in the DLM (Fig. 4A and B). In the trimester averaged model, we also only found that NO_2_ during the second trimester shown significant associations with SGA among girls (Fig. 4C). However, we did not find significant associations between NO_2_ on other birthweight outcomes stratified by children’s sex (SI Table S3 and Figure S4). For PM_2.5_, we did not find consistent children’s sex-specific associations between preconception and gestational PM_2.5_ exposure and birthweight outcomes (SI Table S4 and Figure S5). Our sensitivity analyses confirmed the robustness of the findings from the main analysis as shown above. In the analysis using weekly exposures (as opposed to preconception and trimester-specific exposures), increased NO_2_ and PM_2.5_ exposures during gestational week 12 and gestational week 27 exposure were each significantly associated with increased odds of SGA (SI Figure S6). This is consistent with our main analysis finding that NO_2_ exposure in the 2nd and 3rd trimester and PM_2.5_ exposure in the 3rd trimester was positively associated with increased odds of SGA. Lastly, two-pollutant models did not show significant associations between a pollutant with birthweight outcomes in trimester-averaged models (SI Table S5), only NO_2_ exposure during second trimester demonstrated a marginally significant association with SGA (OR = 1.13 per 10 μg/m^3^ increase, p = 0.06). Lastly, the model assessing the associations between preconception and trimester-averaged air pollution exposure and birthweight, not adjusting for gestational age did not significantly alter the effect estimates and no significantly associations between NO_2_ or PM_2.5_ air pollution exposure on birthweight outcomes were identified (SI Table S6).

## Discussion

In this study, we found that exposure to NO_2_ and PM_2.5_ during gestational period was associated with increased risk of term SGA, and we identified that later pregnancy period including second and third trimester and gestational week 22–32 weeks is one of the critical periods of air pollutant health effects. No significant associations were found between preconception air pollution and birthweight outcomes. Additionally, we found newborn sex-specific associations of NO_2_ exposure on SGA outcome and identified that girls were more susceptible to prenatal air pollution exposure, especially during later pregnancy period.

Our findings on increased risks of adverse birth outcomes associated with gestational air pollution exposure were consistent with previous epidemiologic observations showing that PM_2.5_ and NO_2_ exposures were associated with increased of risk of SGA [12, 18, 43–45]. A systematic review conducted in 2012 [12] summarized from 10 studies that per 20 ppb (equivalent to 37.70 μg/m^3^) increase in NO_2_ exposure was associated with a 28.1 g (95% CI: 11.5 – 44.8 g) decrease in birthweight. Studies published subsequently provided similar supportive results, even though the critical windows in some studies were not same as we found. Le et al. (2012) have shown that among 164905 pregnant women in Detroit, Michigan, exposure to high levels of NO_2_ (> 18.7 ppb, equivalent to 35.1 μg/m^3^) during the first month of the pregnancy was associated with 10% (95% CI: 1 – 19%) increases of odds of SGA [43]. In a cohort of 628 women in Los Angeles, California, Niu et al., (2022) found that 1 IQR increase in PM_2.5_ (4 μg/m^3^) during 4–22 gestational weeks was associated with 9.5 g (95% CI: 8.6–10.4 g) decrease in birthweight, and 1 IQR increase in NO_2_ (11 ppb, equivalent to 5.8 μg/m^3^) from 9 to 14 gestational weeks was associated with 40.4 g (95% CI: 33.3–47.4 g) decrease in birthweight, respectively, and the associations were larger for pregnant women experiencing higher perceived stress. Rachel B Smith et al. (2017) found among half a million pregnant women, per 1 IQR (8.6 μg/m^3^, equivalent to 4.6 ppb) increase in NO_2_ exposure during pregnancy was associated with 3% (95% CI: 1 – 6%) increased in odds of SGA after adjusting for night noise, and the associations were strongest in the second trimester [44].

Similar results were also found in studies in China, showing that later pregnancy was a critical time of air pollution exposure in terms of birthweight outcomes. In a natural experimental design with a month-long air quality improvement during the 2008 Beijing summer Olympics and Paralympics, Rich et al. (2015) found that air pollution exposure in the eighth month of pregnancy was more strongly and significantly associated with decreased birthweight than exposure in other months of pregnancy. Specifically, they reported that 1 IQR increase in PM_2.5_ (19.8 μg/m^3^) and 1 IQR increase in NO_2_ (13.6 ppb) were associated with 18 g (95% CI: 3 – 32 g) and 34 g (95% CI: -3 – 72 g) decrease of birthweight. In the present GAAP study analysis, we found similar associations between NO_2_ and PM_2.5_ exposures and SGA outcomes, but non-significant associations with birthweight. Specifically, we found that exposure to NO_2_ during second and third trimester and exposure to PM_2.5_ during the third trimester were associated with SGA, respectively. Additionally, we identified that gestational weeks 22 to 32 were critical period. We mainly focused on SGA instead of low birthweight (LBW) in this study, this is due to (1) SGA is determined together by birthweight, children’s sex and gestational age at delivery with a reference chart specific for Chinese population [36], (2) LBW is relatively low prevalence and could be influenced by other factors pregnancy complications and preterm birth [46, 47]. The biological mechanisms of gestational air pollution exposure on SGA were not fully elucidated, but possible mechanisms included that exposure to air pollution could affect human placenta physiology [48], alter placenta DNA methylation [49] and suppress antioxidant defense systems [50, 51]. In mid-to-late pregnancy periods, previous studies indicated that air pollution exposure could particularly impact placental mitochondrial DNA content and increase oxidative stress and inflammation [51, 52], leading to the dysregulation of fetal weight gain.

In the two-pollutant model, however, we did not find significant associations between one air pollutant with birthweight outcomes (SI Figure S5). This is probably due to (1) relatively high correlation of NO_2_ and PM_2.5_ (SI Figure S2) and including correlated two variables in the model could lead to unstable estimates, (2) co-exposure confounding between NO_2_ and PM_2.5_. Since NO_2_ has stronger effects than PM_2.5_ in two-pollutant model, it seems to indicate that NO_2_ rather than PM_2.5_ may be the driver of the associations with SGA. In our study, we examined the potential children’s sex-specific associations and found higher estimated risks of SGA among newborn girls than boys (Fig. 4). Our findings on sex-difference are an important contribution to the literature, as only one previous study reported similar findings that newborn girls had a higher risk of SGA after gestational air pollution exposure [18]. Previous reviews and studies suggested that female newborns were more at an increased risk of lower birthweight and lower birthweight z-scores with air pollution exposure [53–55], which are in line with findings of our study on the outcome of SGA. The biological mechanisms behind sex-specific susceptibility remains to be uncovered thoroughly. However, compared to male fetuses, female fetuses have shown to have lower antioxidant responses than male fetuses such as microRNA and protein markers [56], and potential mechanisms underlie the effects of air pollution on SGA included reduction in transplacental oxygen and nutrient transport and the suppression of antioxidant defense system [53, 57]. Therefore, the lower antioxidative capacity of female fetus could partly explain higher susceptibility of female fetus to air pollution exposure.

We did not find significant associations between preconception air pollution exposure and birthweight outcomes. This non-significant associations of preconception air pollution on birthweight outcomes were also reported by most of the previous literatures [22–24]. However, the previous studies indicated the possible mechanisms linking preconception air pollution and children’s birthweight included alternations of epigenetics and DNA methylations in gametes, maternal oxidative stress, disruption of endocrine in gametogenesis, and changing placental mitochondrial DNA content [28, 58, 59]. Current systematic review and meta-analysis did not find a coherent finding of preconception air pollution and children’s birthweight outcome [60]; therefore, more epidemiological studies are warranted to assess causal effects of children’s health effects from preconception air pollution exposure.

Our study has a few strengths. First, this study leveraged a well characterized preconception birth cohort, with information collected during both preconception and gestational period. We collected maternal information and risk factors of birth outcome during both preconception period and gestational period, such as smoking exposure, maternal BMI. Second, we used a high-resolution spatial-temporal air pollution model to assess weekly exposures to NO_2_ and PM_2.5_ during both preconception and pregnancy periods at residential addresses. In addition, we conducted analysis of both trimester-averaged air pollution exposure as well as weekly averages of exposure using DLM, to identify critical time window in both the preconception and the gestational period of air pollution exposure. This study has some limitations that merit improvements in the future. Pregnant women could be exposed to air pollutants from indoor sources such as cooking, workplace, hospital, and other places. Our air pollution exposure model at individual address level does not include those exposure from other sources and will induce bias if pregnant women moved out of their residential address. Second, we do not have paternal information in this study. Since paternal exposure to environmental pollution and smoking could lead to lower birthweight [61, 62], assessing paternal exposure together with maternal exposure could further clarify whether air pollution exposure during preconception period can affect birth outcomes. Some medical conditions such as gestational diabetes, gestational hypertension are also factors affecting birthweight outcomes [63, 64], but not included in this study as covariates. In addition, our cohort has some missing covariates in ethnicity, occupation, and education levels and missingness was treated as a separate category in the analysis. These factors could contribute to the bias when estimating associations between maternal preconception air pollution and birthweight outcomes, potentially leading to the non-significant findings reported in this study. Lastly, this study was only based on population consisting of mostly Han Chinese population in urban or peri-urban areas in Shanghai of relatively high air pollution levels. The findings may not be extrapolated to populations with lower exposure levels.

## Conclusion

We found increased risks for term SGA associated with maternal NO_2_ exposure in the second, third trimester and with maternal PM_2.5_ exposure in the third trimester. The magnitudes of NO_2_ and PM_2.5_ effect estimates were similar to those reported in the existing literature. We identified gestational weeks 22–32, within the entire 52 weeks of peri-conception period consist of 15 weeks of preconception and 37 weeks of gestational period, as a critical time window of exposure affecting birth outcomes in 14220 pregnant women in Shanghai, China. The associations between gestational NO_2_ air pollution exposure and SGA were stronger among newborn girls than boys. No significant associations were found between preconception air pollution exposure and birthweight or birthweight Z-scores, and no associations with LGA, birthweight and birthweight Z-score were found with air pollution exposure during pregnancy. Our novel finding on the sex difference warrants further epidemiologic and mechanistic investigation.

### Electronic supplementary material

Below is the link to the electronic supplementary material.

Supplementary Material 1

## Data Availability

The data described in this article can be accessed upon request after institutional review board (IRB) approvals.
