# Supplementary material for: Identifying critical windows of air pollution exposure during preconception and gestational period on birthweight: a prospective cohort study
Source: Environ Health. 2023 Oct 19;22:71. doi: 10.1186/s12940-023-01022-6 (PMC10585741; doi:10.1186/s12940-023-01022-6)

Supplementary information for

Identifying Critical Windows of Air Pollution Exposure during Preconception and Gestational Period on Birthweight: A Prospective Cohort Study

Jiawen Liao^1#^, Yi Zhang^2#^, Zhenchun Yang^3^, Chenyu Qiu^1^, Wu Chen^1^, Junfeng (Jim) Zhang^3,4^, Kiros Berhane^5^, Zhipeng Bai^6^, Bin Han^6^, Jia Xu^6^, Yong-hui Jiang^7^, Frank Gilliland^1^, Weili Yan^2^, Guoying Huang^2*^ and Zhanghua Chen^1*^

^1^ Department of Population and Public Health Sciences, Keck School of Medicine of University of Southern California, Los Angeles, CA, United States of America

^2^ Children’s Hospital of Fudan University, Shanghai Key Laboratory of Birth Defect, Shanghai, China

^3^ Duke Global Health Institute, Durham, NC, United States of America

^4^ Division of Environmental Science and Policy, Nicholas School of the Environment, Duke University, Durham, NC, United States of America

^5^ Department of Biostatistics, Mailman School of Public Health, Columbia University, New York, NY, United States of America

^6^ State Key Laboratory of Environmental Criteria and Risk Assessment, Chinese Research Academy of Environmental Sciences, Beijing, China

^7^ Department of Genetics, Neuroscience, and Pediatrics, Yale University School of Medicine, New Haven, CT, United States of America

^#^Authors contributed equally to the manuscript

*Corresponding author: Dr. Zhanghua Chen, [zhanghuc@usc.edu](mailto:zhanghuc@usc.edu); and Dr. Guoying Huang: [gyhuang@shmu.edu.cn](mailto:gyhuang@shmu.edu.cn)

**Table S1**. Distributed lag model (DLM) results of weekly NO_2_ air pollution exposure from 15 weeks before conception to 37 weeks of gestation.

|  | Z-score | | | Birthweight (gram) | | | OR of SGA | | | OR of LGA | | |
| --- | --- | --- | --- | --- | --- | --- | --- | --- | --- | --- | --- | --- |
| Gestataional weeks | Point estimate | low | high | point_est | low | high | point_est | low | high | point_est | low | high |
| -15 | 0.000 | -0.008 | 0.008 | 1.166 | -1.946 | 4.278 | 0.992 | 0.957 | 1.028 | 0.997 | 0.973 | 1.022 |
| -14 | 0.000 | -0.007 | 0.007 | 1.087 | -1.804 | 3.978 | 0.992 | 0.959 | 1.026 | 0.997 | 0.974 | 1.021 |
| -13 | 0.000 | -0.007 | 0.007 | 1.008 | -1.666 | 3.683 | 0.992 | 0.962 | 1.023 | 0.997 | 0.976 | 1.019 |
| -12 | 0.000 | -0.006 | 0.006 | 0.930 | -1.533 | 3.393 | 0.992 | 0.965 | 1.021 | 0.997 | 0.978 | 1.017 |
| -11 | 0.000 | -0.006 | 0.006 | 0.852 | -1.406 | 3.110 | 0.993 | 0.967 | 1.019 | 0.997 | 0.979 | 1.016 |
| -10 | 0.000 | -0.006 | 0.005 | 0.775 | -1.287 | 2.838 | 0.993 | 0.970 | 1.017 | 0.997 | 0.981 | 1.014 |
| -9 | 0.000 | -0.005 | 0.004 | 0.699 | -1.178 | 2.577 | 0.993 | 0.972 | 1.015 | 0.997 | 0.982 | 1.013 |
| -8 | 0.000 | -0.005 | 0.004 | 0.624 | -1.082 | 2.331 | 0.993 | 0.974 | 1.013 | 0.998 | 0.984 | 1.011 |
| -7 | 0.000 | -0.004 | 0.003 | 0.550 | -1.001 | 2.102 | 0.994 | 0.976 | 1.012 | 0.998 | 0.985 | 1.010 |
| -6 | -0.001 | -0.004 | 0.003 | 0.478 | -0.938 | 1.895 | 0.994 | 0.978 | 1.010 | 0.998 | 0.986 | 1.009 |
| -5 | -0.001 | -0.004 | 0.003 | 0.408 | -0.898 | 1.713 | 0.994 | 0.979 | 1.009 | 0.998 | 0.987 | 1.008 |
| -4 | -0.001 | -0.004 | 0.002 | 0.339 | -0.882 | 1.560 | 0.995 | 0.981 | 1.009 | 0.998 | 0.988 | 1.008 |
| -3 | -0.001 | -0.004 | 0.002 | 0.272 | -0.894 | 1.438 | 0.995 | 0.982 | 1.008 | 0.998 | 0.988 | 1.007 |
| -2 | -0.001 | -0.004 | 0.002 | 0.208 | -0.932 | 1.348 | 0.995 | 0.982 | 1.008 | 0.998 | 0.989 | 1.007 |
| -1 | -0.001 | -0.004 | 0.002 | 0.146 | -0.996 | 1.288 | 0.996 | 0.983 | 1.009 | 0.998 | 0.989 | 1.007 |
| 0 | -0.001 | -0.004 | 0.002 | 0.086 | -1.080 | 1.253 | 0.996 | 0.983 | 1.009 | 0.998 | 0.989 | 1.008 |
| 1 | -0.001 | -0.004 | 0.002 | 0.030 | -1.179 | 1.238 | 0.996 | 0.983 | 1.010 | 0.998 | 0.989 | 1.008 |
| 2 | -0.001 | -0.004 | 0.002 | -0.024 | -1.285 | 1.237 | 0.997 | 0.982 | 1.011 | 0.998 | 0.988 | 1.009 |
| 3 | -0.001 | -0.004 | 0.002 | -0.075 | -1.395 | 1.245 | 0.997 | 0.982 | 1.012 | 0.999 | 0.988 | 1.009 |
| 4 | -0.001 | -0.005 | 0.003 | -0.122 | -1.502 | 1.258 | 0.998 | 0.982 | 1.014 | 0.999 | 0.988 | 1.010 |
| 5 | -0.001 | -0.005 | 0.003 | -0.166 | -1.604 | 1.272 | 0.998 | 0.982 | 1.015 | 0.999 | 0.987 | 1.011 |
| 6 | -0.001 | -0.005 | 0.003 | -0.206 | -1.697 | 1.285 | 0.999 | 0.982 | 1.016 | 0.999 | 0.987 | 1.011 |
| 7 | -0.001 | -0.005 | 0.003 | -0.242 | -1.778 | 1.294 | 0.999 | 0.982 | 1.017 | 0.999 | 0.987 | 1.012 |
| 8 | -0.001 | -0.005 | 0.003 | -0.274 | -1.846 | 1.297 | 1.000 | 0.982 | 1.018 | 0.999 | 0.987 | 1.012 |
| 9 | -0.001 | -0.005 | 0.003 | -0.302 | -1.898 | 1.294 | 1.001 | 0.983 | 1.019 | 1.000 | 0.987 | 1.013 |
| 10 | -0.001 | -0.005 | 0.003 | -0.326 | -1.934 | 1.283 | 1.001 | 0.983 | 1.020 | 1.000 | 0.987 | 1.013 |
| 11 | -0.001 | -0.005 | 0.003 | -0.345 | -1.952 | 1.263 | 1.002 | 0.984 | 1.021 | 1.000 | 0.987 | 1.013 |
| 12 | -0.001 | -0.005 | 0.003 | -0.359 | -1.953 | 1.235 | 1.003 | 0.985 | 1.021 | 1.000 | 0.988 | 1.013 |
| 13 | -0.001 | -0.005 | 0.003 | -0.369 | -1.938 | 1.200 | 1.004 | 0.986 | 1.022 | 1.001 | 0.988 | 1.013 |
| 14 | -0.001 | -0.005 | 0.003 | -0.375 | -1.909 | 1.158 | 1.005 | 0.987 | 1.022 | 1.001 | 0.989 | 1.013 |
| 15 | -0.001 | -0.005 | 0.003 | -0.378 | -1.866 | 1.110 | 1.005 | 0.989 | 1.023 | 1.001 | 0.989 | 1.013 |
| 16 | -0.001 | -0.004 | 0.003 | -0.376 | -1.811 | 1.059 | 1.006 | 0.990 | 1.023 | 1.002 | 0.990 | 1.013 |
| 17 | -0.001 | -0.004 | 0.003 | -0.371 | -1.747 | 1.005 | 1.007 | 0.992 | 1.023 | 1.002 | 0.991 | 1.013 |
| 18 | -0.001 | -0.004 | 0.003 | -0.363 | -1.677 | 0.952 | 1.008 | 0.993 | 1.023 | 1.002 | 0.992 | 1.013 |
| 19 | -0.001 | -0.004 | 0.003 | -0.351 | -1.604 | 0.902 | 1.009 | 0.995 | 1.024 | 1.003 | 0.993 | 1.013 |
| 20 | 0.000 | -0.004 | 0.003 | -0.336 | -1.533 | 0.860 | 1.010 | 0.997 | 1.024 | 1.003 | 0.993 | 1.013 |
| 21 | 0.000 | -0.003 | 0.003 | -0.319 | -1.468 | 0.830 | 1.011 | 0.998 | 1.025 | 1.003 | 0.994 | 1.013 |
| 22 | 0.000 | -0.003 | 0.003 | -0.299 | -1.415 | 0.818 | **1.013** | **1.000** | **1.025** | 1.004 | 0.995 | 1.013 |
| 23 | 0.000 | -0.003 | 0.003 | -0.276 | -1.381 | 0.829 | **1.014** | **1.001** | **1.026** | 1.004 | 0.995 | 1.013 |
| 24 | 0.000 | -0.003 | 0.003 | -0.251 | -1.369 | 0.867 | **1.015** | **1.002** | **1.028** | 1.004 | 0.995 | 1.014 |
| 25 | 0.000 | -0.003 | 0.003 | -0.224 | -1.384 | 0.936 | **1.016** | **1.003** | **1.029** | 1.005 | 0.996 | 1.014 |
| 26 | 0.000 | -0.003 | 0.003 | -0.195 | -1.426 | 1.036 | **1.017** | **1.003** | **1.031** | 1.005 | 0.995 | 1.015 |
| 27 | 0.000 | -0.003 | 0.004 | -0.164 | -1.493 | 1.165 | **1.018** | **1.003** | **1.034** | 1.006 | 0.995 | 1.016 |
| 28 | 0.000 | -0.003 | 0.004 | -0.132 | -1.583 | 1.320 | **1.020** | **1.003** | **1.036** | 1.006 | 0.994 | 1.018 |
| 29 | 0.000 | -0.004 | 0.004 | -0.098 | -1.692 | 1.496 | **1.021** | **1.003** | **1.039** | 1.007 | 0.994 | 1.019 |
| 30 | 0.000 | -0.004 | 0.005 | -0.063 | -1.816 | 1.691 | **1.022** | **1.002** | **1.042** | 1.007 | 0.993 | 1.021 |
| 31 | 0.001 | -0.004 | 0.005 | -0.027 | -1.954 | 1.901 | **1.023** | **1.001** | **1.046** | 1.007 | 0.992 | 1.023 |
| 32 | 0.001 | -0.005 | 0.006 | 0.010 | -2.102 | 2.123 | **1.025** | **1.000** | **1.049** | 1.008 | 0.991 | 1.025 |
| 33 | 0.001 | -0.005 | 0.007 | 0.048 | -2.258 | 2.354 | 1.026 | 0.999 | 1.053 | 1.008 | 0.990 | 1.027 |
| 34 | 0.001 | -0.006 | 0.007 | 0.086 | -2.421 | 2.594 | 1.027 | 0.998 | 1.057 | 1.009 | 0.989 | 1.029 |
| 35 | 0.001 | -0.006 | 0.008 | 0.125 | -2.589 | 2.839 | 1.028 | 0.997 | 1.061 | 1.009 | 0.987 | 1.031 |
| 36 | 0.001 | -0.006 | 0.009 | 0.164 | -2.760 | 3.088 | 1.030 | 0.996 | 1.064 | 1.010 | 0.986 | 1.034 |

**Table S2**. Distributed lag model (DLM) results of weekly PM_2.5_ air pollution exposure from 15 weeks before conception to 37 weeks of gestation.

|  | Z-score | | | Birthweight (gram) | | | OR of SGA | | | OR of LGA | | |
| --- | --- | --- | --- | --- | --- | --- | --- | --- | --- | --- | --- | --- |
| Gestataional weeks | Point estimate | low | high | point_est | low | high | point_est | low | high | point_est | low | high |
| -15 | 0.000 | -0.011 | 0.011 | 1.902 | -2.060 | 5.865 | 1.002 | 0.957 | 1.049 | 1.007 | 0.976 | 1.040 |
| -14 | 0.000 | -0.010 | 0.011 | 1.848 | -1.870 | 5.566 | 1.001 | 0.959 | 1.046 | 1.007 | 0.977 | 1.038 |
| -13 | 0.000 | -0.009 | 0.010 | 1.794 | -1.687 | 5.274 | 1.001 | 0.961 | 1.042 | 1.007 | 0.979 | 1.036 |
| -12 | 0.001 | -0.008 | 0.009 | 1.739 | -1.512 | 4.991 | 1.001 | 0.964 | 1.039 | 1.007 | 0.981 | 1.033 |
| -11 | 0.001 | -0.007 | 0.009 | 1.685 | -1.347 | 4.717 | 1.000 | 0.966 | 1.036 | 1.007 | 0.982 | 1.031 |
| -10 | 0.001 | -0.006 | 0.008 | 1.632 | -1.194 | 4.457 | 1.000 | 0.968 | 1.033 | 1.007 | 0.984 | 1.029 |
| -9 | 0.001 | -0.006 | 0.008 | 1.579 | -1.055 | 4.212 | 1.000 | 0.970 | 1.031 | 1.007 | 0.985 | 1.028 |
| -8 | 0.001 | -0.005 | 0.007 | 1.526 | -0.933 | 3.984 | 0.999 | 0.971 | 1.028 | 1.007 | 0.986 | 1.026 |
| -7 | 0.001 | -0.005 | 0.007 | 1.474 | -0.830 | 3.778 | 0.999 | 0.973 | 1.026 | 1.007 | 0.987 | 1.025 |
| -6 | 0.001 | -0.004 | 0.007 | 1.422 | -0.749 | 3.594 | 0.999 | 0.974 | 1.024 | 1.007 | 0.988 | 1.023 |
| -5 | 0.001 | -0.004 | 0.007 | 1.372 | -0.693 | 3.437 | 0.999 | 0.975 | 1.023 | 1.007 | 0.989 | 1.022 |
| -4 | 0.001 | -0.004 | 0.007 | 1.322 | -0.662 | 3.306 | 0.998 | 0.976 | 1.021 | 1.007 | 0.989 | 1.022 |
| -3 | 0.002 | -0.003 | 0.007 | 1.273 | -0.657 | 3.204 | 0.998 | 0.976 | 1.020 | 1.007 | 0.990 | 1.021 |
| -2 | 0.002 | -0.003 | 0.007 | 1.226 | -0.677 | 3.128 | 0.998 | 0.976 | 1.020 | 1.007 | 0.990 | 1.021 |
| -1 | 0.002 | -0.003 | 0.007 | 1.179 | -0.720 | 3.078 | 0.998 | 0.976 | 1.020 | 1.007 | 0.990 | 1.020 |
| 0 | 0.002 | -0.003 | 0.007 | 1.134 | -0.782 | 3.050 | 0.998 | 0.976 | 1.020 | 1.007 | 0.989 | 1.021 |
| 1 | 0.002 | -0.003 | 0.007 | 1.090 | -0.860 | 3.040 | 0.998 | 0.976 | 1.020 | 1.007 | 0.989 | 1.021 |
| 2 | 0.002 | -0.003 | 0.007 | 1.047 | -0.948 | 3.043 | 0.998 | 0.975 | 1.021 | 1.007 | 0.989 | 1.021 |
| 3 | 0.002 | -0.004 | 0.007 | 1.006 | -1.043 | 3.055 | 0.998 | 0.975 | 1.021 | 1.007 | 0.988 | 1.021 |
| 4 | 0.002 | -0.004 | 0.008 | 0.967 | -1.140 | 3.073 | 0.998 | 0.974 | 1.022 | 1.007 | 0.988 | 1.022 |
| 5 | 0.002 | -0.004 | 0.008 | 0.929 | -1.236 | 3.093 | 0.998 | 0.973 | 1.023 | 1.007 | 0.987 | 1.022 |
| 6 | 0.002 | -0.004 | 0.008 | 0.893 | -1.327 | 3.113 | 0.998 | 0.973 | 1.024 | 1.007 | 0.987 | 1.023 |
| 7 | 0.002 | -0.004 | 0.008 | 0.858 | -1.412 | 3.128 | 0.998 | 0.973 | 1.025 | 1.007 | 0.987 | 1.023 |
| 8 | 0.002 | -0.004 | 0.008 | 0.826 | -1.487 | 3.139 | 0.999 | 0.972 | 1.025 | 1.007 | 0.986 | 1.024 |
| 9 | 0.002 | -0.004 | 0.008 | 0.796 | -1.551 | 3.142 | 0.999 | 0.972 | 1.026 | 1.007 | 0.986 | 1.024 |
| 10 | 0.002 | -0.004 | 0.008 | 0.767 | -1.602 | 3.136 | 0.999 | 0.973 | 1.027 | 1.007 | 0.986 | 1.025 |
| 11 | 0.002 | -0.004 | 0.008 | 0.741 | -1.639 | 3.121 | 1.000 | 0.973 | 1.028 | 1.007 | 0.986 | 1.025 |
| 12 | 0.002 | -0.004 | 0.008 | 0.717 | -1.662 | 3.096 | 1.000 | 0.973 | 1.028 | 1.007 | 0.986 | 1.025 |
| 13 | 0.002 | -0.004 | 0.008 | 0.695 | -1.673 | 3.063 | 1.001 | 0.974 | 1.029 | 1.007 | 0.987 | 1.025 |
| 14 | 0.002 | -0.003 | 0.008 | 0.675 | -1.671 | 3.021 | 1.002 | 0.975 | 1.029 | 1.007 | 0.987 | 1.025 |
| 15 | 0.002 | -0.003 | 0.008 | 0.657 | -1.658 | 2.972 | 1.002 | 0.976 | 1.030 | 1.007 | 0.988 | 1.025 |
| 16 | 0.002 | -0.003 | 0.008 | 0.640 | -1.636 | 2.917 | 1.003 | 0.977 | 1.030 | 1.007 | 0.988 | 1.025 |
| 17 | 0.002 | -0.003 | 0.008 | 0.626 | -1.606 | 2.857 | 1.004 | 0.979 | 1.030 | 1.007 | 0.989 | 1.025 |
| 18 | 0.002 | -0.003 | 0.008 | 0.612 | -1.570 | 2.795 | 1.005 | 0.980 | 1.031 | 1.007 | 0.990 | 1.025 |
| 19 | 0.002 | -0.003 | 0.007 | 0.601 | -1.530 | 2.731 | 1.006 | 0.981 | 1.031 | 1.007 | 0.991 | 1.025 |
| 20 | 0.002 | -0.003 | 0.007 | 0.590 | -1.489 | 2.670 | 1.007 | 0.983 | 1.031 | 1.007 | 0.991 | 1.025 |
| 21 | 0.002 | -0.003 | 0.007 | 0.581 | -1.449 | 2.612 | 1.008 | 0.985 | 1.032 | 1.007 | 0.992 | 1.025 |
| 22 | 0.002 | -0.003 | 0.007 | 0.574 | -1.415 | 2.562 | 1.009 | 0.986 | 1.032 | 1.007 | 0.993 | 1.025 |
| 23 | 0.002 | -0.003 | 0.007 | 0.567 | -1.388 | 2.523 | 1.010 | 0.988 | 1.033 | 1.007 | 0.994 | 1.025 |
| 24 | 0.002 | -0.003 | 0.007 | 0.562 | -1.373 | 2.497 | 1.011 | 0.989 | 1.034 | 1.007 | 0.994 | 1.026 |
| 25 | 0.002 | -0.003 | 0.007 | 0.558 | -1.372 | 2.487 | 1.012 | 0.990 | 1.035 | 1.007 | 0.995 | 1.026 |
| 26 | 0.002 | -0.003 | 0.007 | 0.554 | -1.389 | 2.497 | 1.013 | 0.991 | 1.036 | 1.007 | 0.995 | 1.027 |
| 27 | 0.002 | -0.003 | 0.007 | 0.552 | -1.425 | 2.528 | 1.015 | 0.992 | 1.038 | 1.007 | 0.995 | 1.027 |
| 28 | 0.002 | -0.003 | 0.007 | 0.550 | -1.482 | 2.582 | 1.016 | 0.993 | 1.040 | 1.007 | 0.995 | 1.028 |
| 29 | 0.002 | -0.003 | 0.007 | 0.549 | -1.560 | 2.657 | 1.017 | 0.993 | 1.042 | 1.007 | 0.995 | 1.029 |
| 30 | 0.002 | -0.004 | 0.007 | 0.548 | -1.658 | 2.754 | 1.018 | 0.993 | 1.044 | 1.007 | 0.995 | 1.031 |
| 31 | 0.002 | -0.004 | 0.008 | 0.548 | -1.774 | 2.870 | 1.020 | 0.993 | 1.047 | 1.007 | 0.995 | 1.032 |
| 32 | 0.002 | -0.004 | 0.008 | 0.548 | -1.908 | 3.005 | 1.021 | 0.993 | 1.050 | 1.007 | 0.994 | 1.034 |
| 33 | 0.002 | -0.005 | 0.008 | 0.549 | -2.057 | 3.155 | 1.022 | 0.993 | 1.053 | 1.007 | 0.993 | 1.036 |
| 34 | 0.002 | -0.005 | 0.009 | 0.550 | -2.218 | 3.318 | 1.024 | 0.992 | 1.056 | 1.007 | 0.993 | 1.038 |
| 35 | 0.002 | -0.005 | 0.009 | 0.551 | -2.391 | 3.493 | 1.025 | 0.992 | 1.060 | 1.007 | 0.992 | 1.040 |
| 36 | 0.002 | -0.006 | 0.009 | 0.552 | -2.573 | 3.677 | 1.026 | 0.991 | 1.063 | 1.007 | 0.991 | 1.042 |

**Table S3.** Stratified model of birthweight (g), Z-score and OR of SGA, LGA and LBW per 1 standard deviation (SD) increase of NO_2_ air pollution (11.5 µg/m^3^) in preconception and pregnancy trimester for full-term birth children.

|  |  | Female | | | | Male | | | |
| --- | --- | --- | --- | --- | --- | --- | --- | --- | --- |
|  |  | Point Estimate | P value | LCI | UCI | Point Estimate | P value | LCI | UCI |
| Birthweight | PreConception | 9.86 | 0.18 | -4.41 | 24.13 | -8.28 | 0.23 | -21.68 | 5.13 |
|  | 1st Trimester | 1.60 | 0.83 | -13.22 | 16.42 | -11.66 | 0.08 | -24.87 | 1.56 |
|  | 2nd Trimester | -5.14 | 0.50 | -20.17 | 9.89 | -8.01 | 0.25 | -21.67 | 5.66 |
|  | 3rd Trimester | 5.31 | 0.54 | -11.61 | 22.23 | -4.67 | 0.55 | -20.02 | 10.68 |
| Z-score | PreConception | 0.023 | 0.227 | -0.014 | 0.061 | -0.021 | 0.234 | -0.055 | 0.013 |
|  | 1st Trimester | 0.003 | 0.883 | -0.036 | 0.042 | -0.028 | 0.101 | -0.062 | 0.005 |
|  | 2nd Trimester | -0.008 | 0.688 | -0.048 | 0.031 | -0.011 | 0.520 | -0.046 | 0.023 |
|  | 3rd Trimester | 0.017 | 0.465 | -0.028 | 0.061 | -0.011 | 0.595 | -0.050 | 0.028 |
|  |  | OR | P value | LCI OR | UCI OR | OR | P value | LCI OR | UCI OR |
| SGA | PreConception | 0.99 | 0.95 | 0.85 | 1.16 | 1.06 | 0.46 | 0.91 | 1.22 |
|  | 1st Trimester | 1.05 | 0.51 | 0.90 | 1.24 | 0.92 | 0.26 | 0.80 | 1.06 |
|  | 2nd Trimester | **1.19** | **0.03** | **1.02** | **1.40** | 1.09 | 0.23 | 0.94 | 1.27 |
|  | 3rd Trimester | 1.15 | 0.16 | 0.95 | 1.38 | 1.14 | 0.12 | 0.97 | 1.36 |
| LGA | PreConception | 1.06 | 0.29 | 0.95 | 1.19 | 0.93 | 0.16 | 0.84 | 1.03 |
|  | 1st Trimester | 1.03 | 0.59 | 0.92 | 1.16 | 0.94 | 0.25 | 0.86 | 1.04 |
|  | 2nd Trimester | 1.01 | 0.90 | 0.90 | 1.13 | 1.06 | 0.30 | 0.95 | 1.17 |
|  | 3rd Trimester | 1.04 | 0.56 | 0.91 | 1.19 | 1.02 | 0.69 | 0.91 | 1.15 |

**Table S4.** Stratified model of birthweight (g), Z-score and OR of SGA, LGA and LBW per 1 SD increase of PM_2.5_ air pollution (9.6 µg/m^3^) in preconception and pregnancy trimester for full-term birth children.

|  |  | Female | | | | Male | | | |
| --- | --- | --- | --- | --- | --- | --- | --- | --- | --- |
|  |  | Point Estimate | P value | LCI | UCI | Point Estimate | P value | LCI | UCI |
| Birthweight | Preconception | **19.77** | **0.04** | **0.69** | **38.86** | 1.32 | 0.88 | -16.17 | 18.81 |
|  | 1st Trimester | 5.09 | 0.56 | -11.93 | 22.11 | -10.04 | 0.18 | -24.83 | 4.75 |
|  | 2nd Trimester | -4.65 | 0.61 | -22.39 | 13.09 | -2.01 | 0.80 | -17.44 | 13.43 |
|  | 3rd Trimester | 3.36 | 0.71 | -14.35 | 21.06 | -2.38 | 0.76 | -17.71 | 12.96 |
| Z-score | PreConception | 0.041 | 0.112 | -0.010 | 0.091 | 0.003 | 0.907 | -0.042 | 0.047 |
|  | 1st Trimester | 0.008 | 0.715 | -0.036 | 0.053 | -0.022 | 0.248 | -0.060 | 0.015 |
|  | 2nd Trimester | -0.005 | 0.848 | -0.051 | 0.042 | 0.010 | 0.624 | -0.029 | 0.049 |
|  | 3rd Trimester | 0.015 | 0.539 | -0.032 | 0.061 | -0.002 | 0.926 | -0.041 | 0.037 |
|  |  | OR | P value | LCI OR | UCI OR | OR | P value | LCI OR | UCI OR |
| SGA | Preconception | 0.87 | 0.19 | 0.70 | 1.07 | 1.07 | 0.52 | 0.88 | 1.30 |
|  | 1st Trimester | 0.91 | 0.31 | 0.75 | 1.09 | 0.95 | 0.51 | 0.81 | 1.11 |
|  | 2nd Trimester | 1.17 | 0.12 | 0.96 | 1.42 | 1.03 | 0.70 | 0.87 | 1.22 |
|  | 3rd Trimester | 1.17 | 0.13 | 0.96 | 1.43 | 1.15 | 0.14 | 0.96 | 1.37 |
| LGA | Preconception | 1.14 | 0.11 | 0.97 | 1.33 | 0.94 | 0.36 | 0.82 | 1.07 |
|  | 1st Trimester | 1.03 | 0.65 | 0.90 | 1.19 | **0.88** | **0.04** | **0.79** | **0.99** |
|  | 2nd Trimester | 1.05 | 0.54 | 0.91 | 1.21 | 1.04 | 0.56 | 0.92 | 1.17 |
|  | 3rd Trimester | 1.10 | 0.22 | 0.95 | 1.27 | 1.02 | 0.79 | 0.90 | 1.15 |

**Table S5**. Two-pollutant model of birthweight (g), Z-score and OR of SGA, LGA and LBW per 1 SD change of air pollution (9.6 µg/m^3^ for PM_2.5_ and 11.5 µg/m^3^ for NO_2_) in preconception and pregnancy trimester for full-term birth children.

|  |  | NO_2_ | | | | PM_2.5_ | | | |
| --- | --- | --- | --- | --- | --- | --- | --- | --- | --- |
|  |  | Point Estimate | P value | LCI | UCI | Point Estimate | P value | LCI | UCI |
| Birthweight | Preconception | -4.92 | 0.41 | -16.60 | 6.76 | 13.07 | 0.09 | -2.34 | 28.48 |
|  | 1st Trimester | -5.59 | 0.36 | -17.53 | 6.35 | -0.50 | 0.94 | -14.04 | 13.04 |
|  | 2nd Trimester | -7.37 | 0.25 | -19.88 | 5.14 | 1.80 | 0.81 | -12.61 | 16.20 |
|  | 3rd Trimester | 0.53 | 0.94 | -13.57 | 14.63 | -0.49 | 0.95 | -14.87 | 13.89 |
|  | Whole pregnancy | -3.14 | 0.51 | -12.42 | 6.14 | -0.58 | 0.69 | -3.37 | 2.21 |
| Z-score | Preconception | -0.009 | 0.543 | -0.039 | 0.021 | 0.027 | 0.177 | -0.012 | 0.067 |
|  | 1st Trimester | -0.012 | 0.439 | -0.043 | 0.019 | -0.001 | 0.938 | -0.036 | 0.034 |
|  | 2nd Trimester | -0.015 | 0.348 | -0.048 | 0.017 | 0.015 | 0.431 | -0.022 | 0.052 |
|  | 3rd Trimester | 0.001 | 0.950 | -0.035 | 0.038 | 0.004 | 0.817 | -0.033 | 0.041 |
|  | Whole pregnancy | -0.008 | 0.520 | -0.032 | 0.016 | 0.001 | 0.750 | -0.006 | 0.008 |
|  |  | OR | P value | LCI OR | UCI OR | OR | P value | LCI OR | UCI OR |
| SGA | Preconception | 1.05 | 0.45 | 0.93 | 1.19 | 0.94 | 0.44 | 0.79 | 1.11 |
|  | 1st Trimester | 1.02 | 0.81 | 0.90 | 1.15 | 0.92 | 0.28 | 0.80 | 1.07 |
|  | 2nd Trimester | 1.13 | 0.06 | 0.99 | 1.29 | 1.00 | 0.99 | 0.86 | 1.17 |
|  | 3rd Trimester | 1.08 | 0.31 | 0.93 | 1.26 | 1.10 | 0.27 | 0.93 | 1.29 |
|  | Whole pregnancy | 1.03 | 0.54 | 0.94 | 1.12 | 1.01 | 0.49 | 0.98 | 1.04 |
| LGA | Preconception | 0.98 | 0.64 | 0.90 | 1.07 | 1.04 | 0.54 | 0.92 | 1.17 |
|  | 1st Trimester | 1.02 | 0.71 | 0.93 | 1.11 | 0.94 | 0.22 | 0.84 | 1.04 |
|  | 2nd Trimester | 1.03 | 0.51 | 0.94 | 1.13 | 1.02 | 0.69 | 0.92 | 1.14 |
|  | 3rd Trimester | 1.02 | 0.75 | 0.91 | 1.13 | 1.04 | 0.48 | 0.93 | 1.16 |
|  | Whole pregnancy | 1.02 | 0.46 | 0.96 | 1.09 | 1.00 | 0.92 | 0.98 | 1.02 |

**Table S6**. Differences in birthweight (g) per 1 standard deviation (SD) increase in preconception and trimester averaged NO_2_ and PM_2.5_ exposure (9.6 µg/m^3^ for PM_2.5_ and 11.5 for NO_2_), respectively. Models are controlled for same covariates as main analysis except for gestational age.

|  |  | NO_2_ | | | | PM_2.5_ | | | |
| --- | --- | --- | --- | --- | --- | --- | --- | --- | --- |
| Outcome | Time period | Point Estimate | P value | LCI | UCI | Point Estimate | P value | LCI | UCI |
| Birthweight | Preconception | 1.71 | 0.75 | -8.66 | 12.07 | 6.05 | 0.39 | -7.60 | 19.70 |
|  | 1st Trimester | -3.8 | 0.48 | -14.19 | 6.69 | -3.05 | 0.61 | -14.89 | 8.78 |
|  | 2nd Trimester | 1.46 | 0.79 | -9.25 | 12.16 | 8.93 | 0.16 | -3.38 | 21.24 |
|  | 3rd Trimester | 1.33 | 0.83 | -10.73 | 13.39 | 2.80 | 0.66 | -9.49 | 15.09 |
|  | Whole pregnancy | -0.42 | 0.92 | -8.99 | 8.15 | 4.35 | 0.39 | -5.47 | 14.16 |

**Figure S1**. Exposure Response Function Curve in Distributed Lag Non-linear Model (DLNM) Comparing Linear, Quadratic Polynomial and Cubic Polynomial Functions.


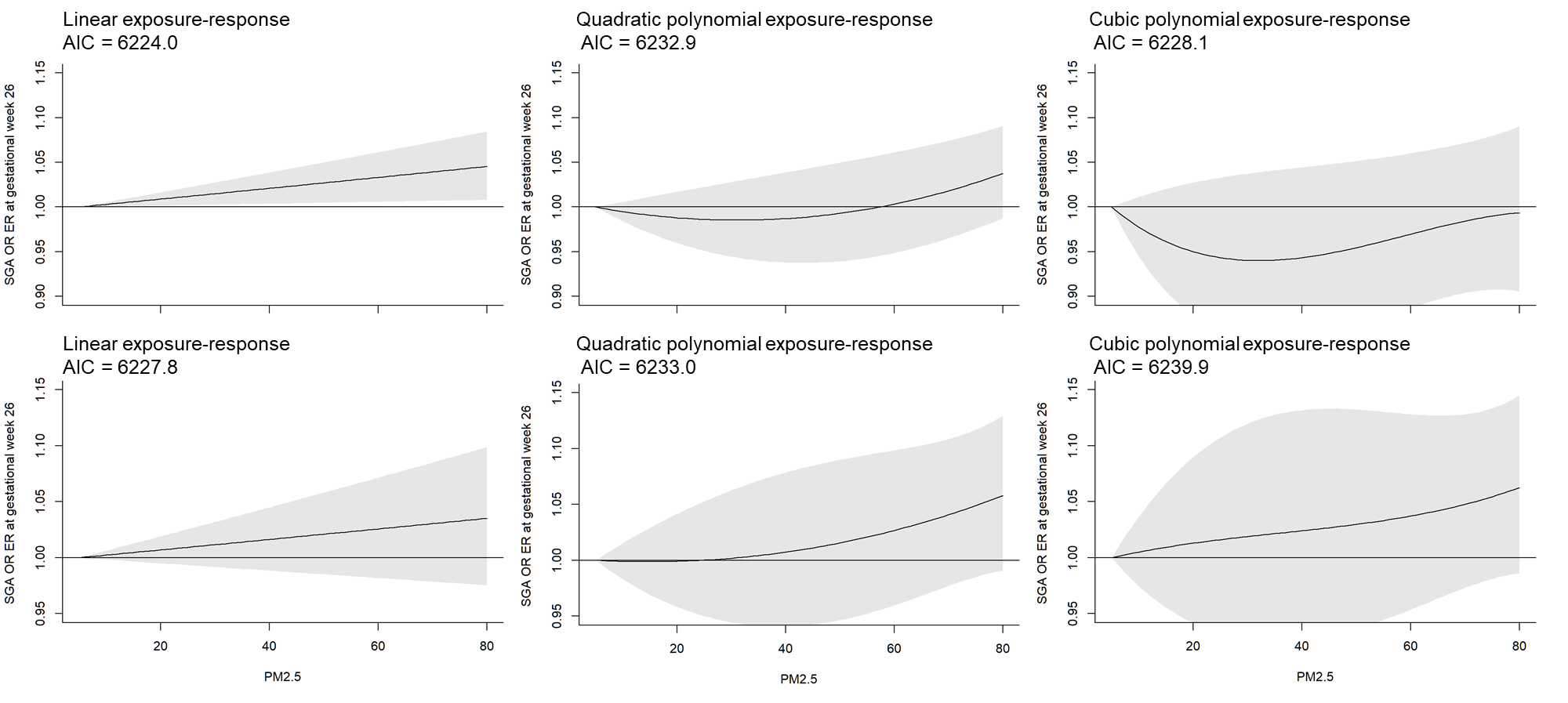


**Figure S2.** Spearman correlation coefficient of air pollution exposure during 3-month pre-conception and each trimester for NO_2_ and PM_2.5_ pollutants. PreC: 3-month preconception period, Tri1: first trimester of pregnancy, Tri2: second trimester of pregnancy, Tri3: third trimester of pregnancy.


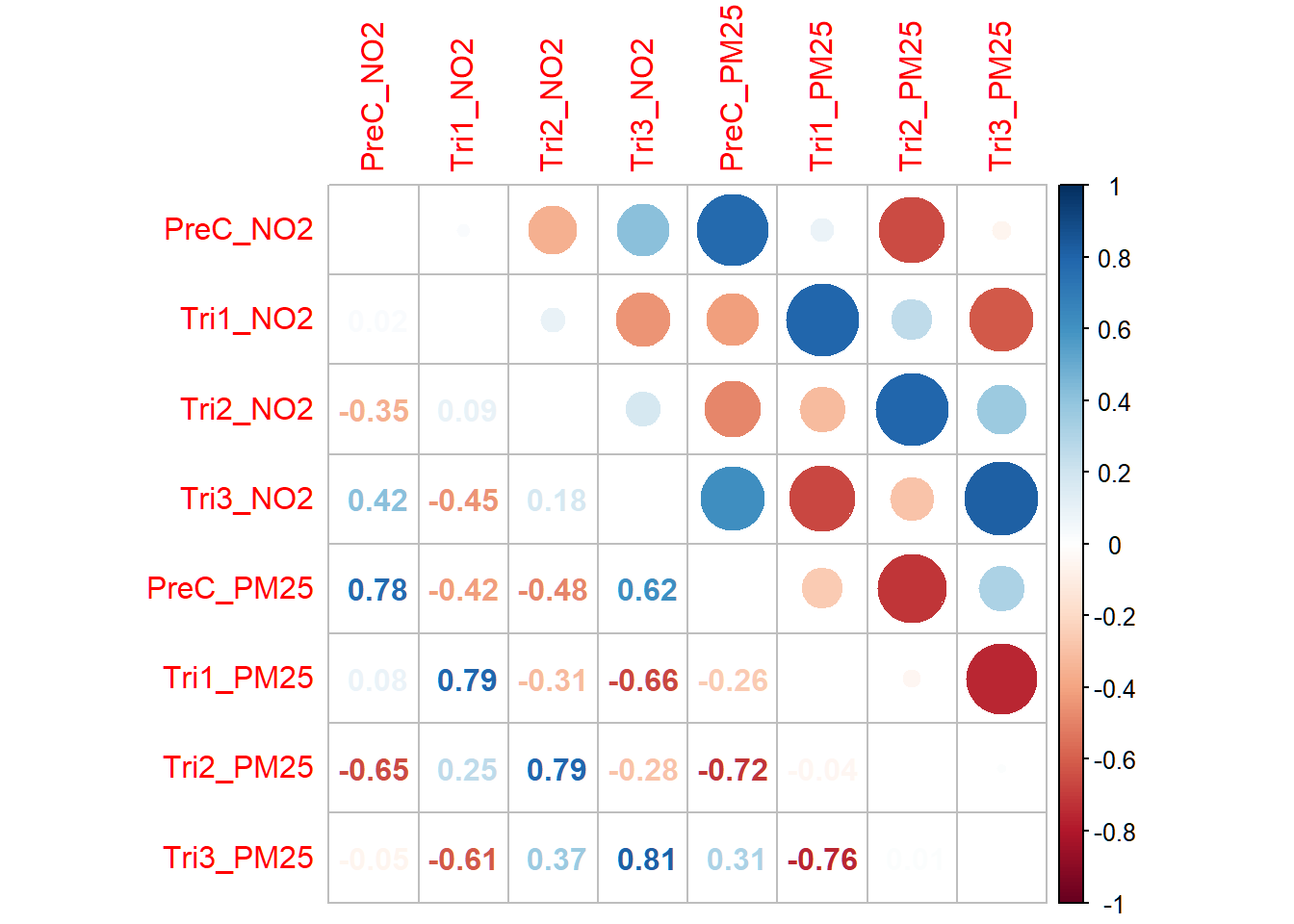


**Figure S3**. Density plot of the NO_2_ and PM_2.5_ air pollutants exposure during 3-month preconception and each trimester period. PreC: 3-month preconception period, Tri1: first trimester, Tri2: second trimester, Tri3: third trimester.


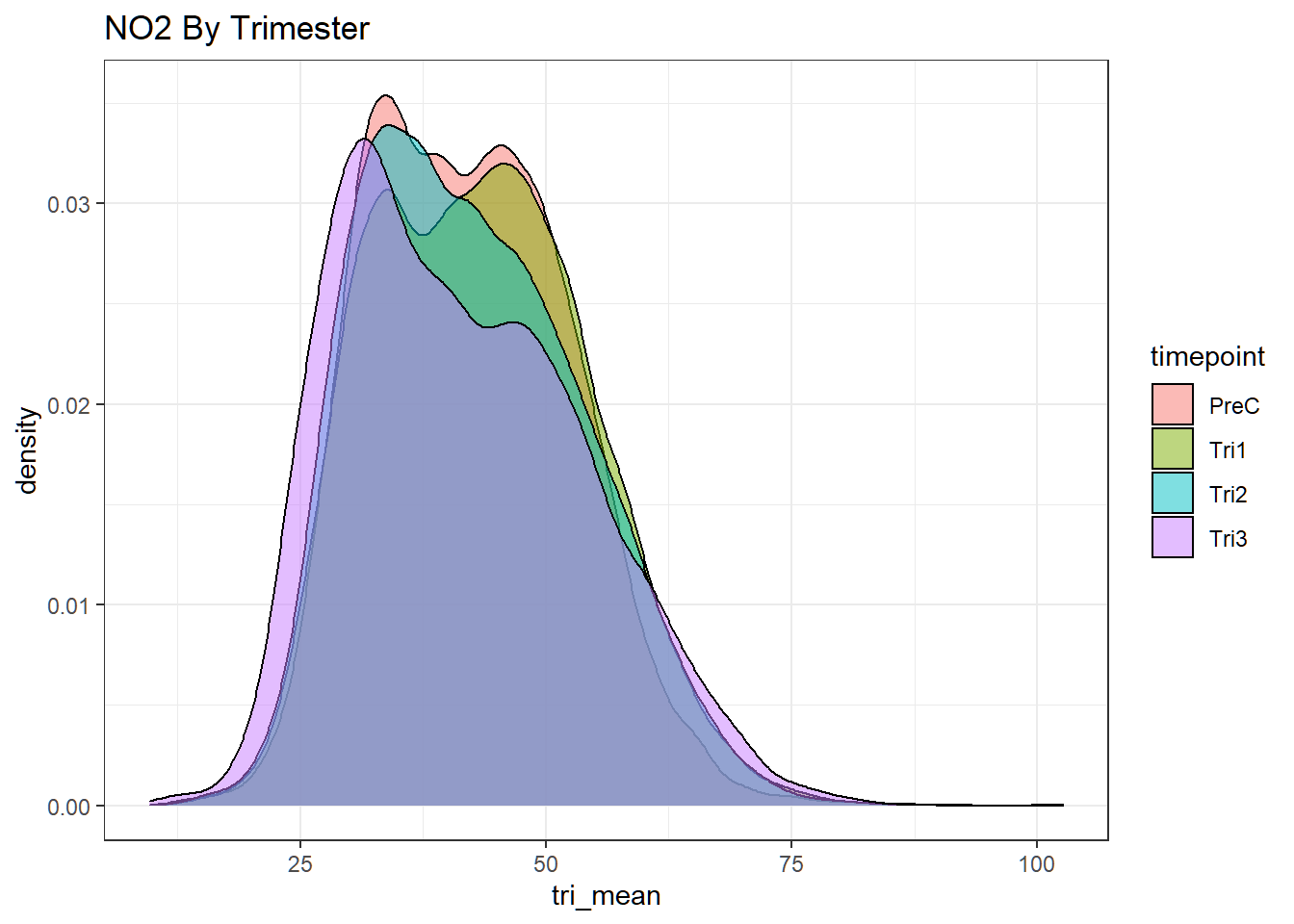

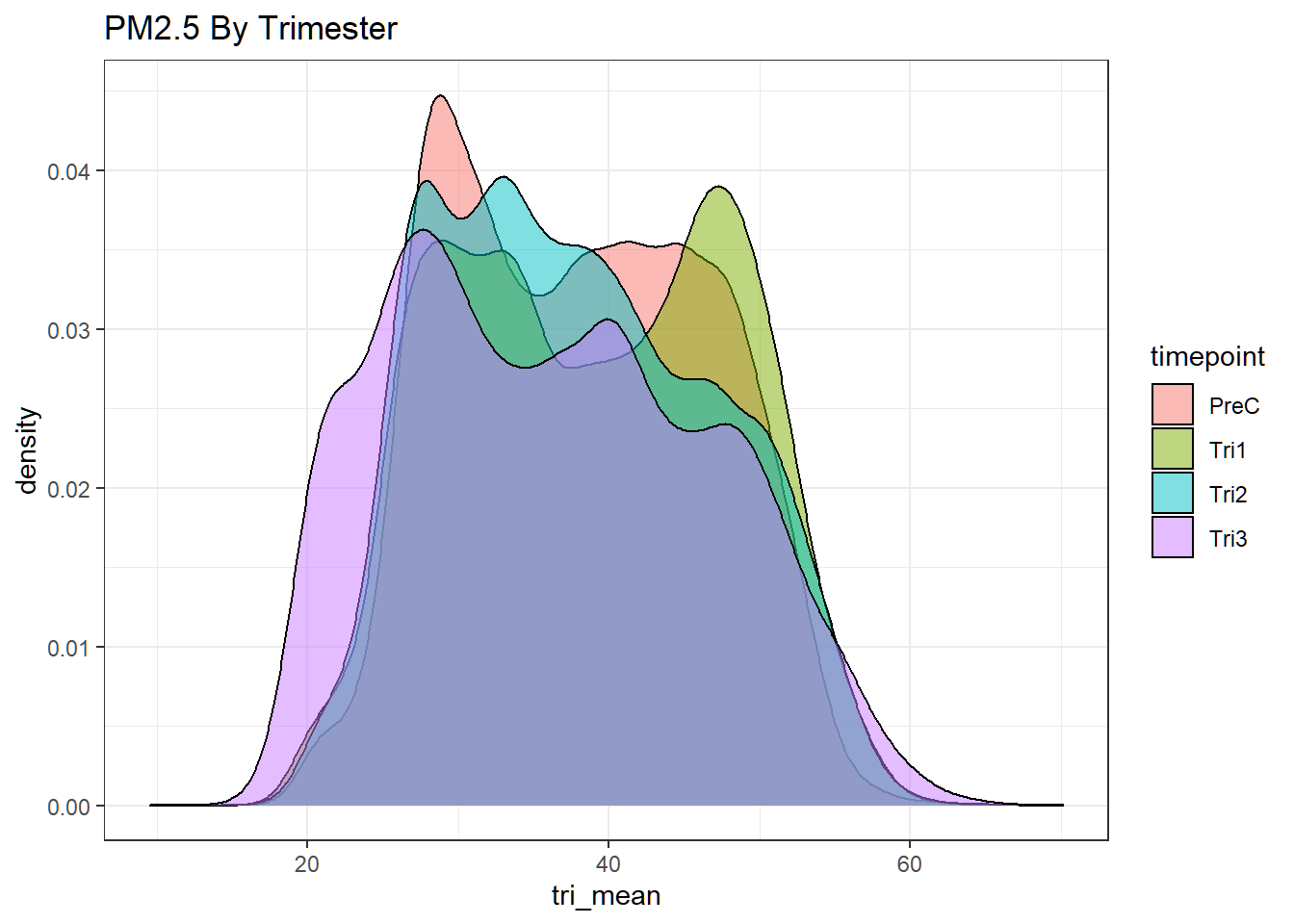


**Figure S4.** Plots of the differences in birthweight Z-score, birthweight, SGA and LGA associated with 1 IQR increase (17.4 µg/m^3^, equivalent to 9.26 ppb) of NO_2_ concentrations during preconception and gestational periods stratified by children’s sex (A: female, B: male). The x-axis indicated the time in week of gestation (i.e. conception date is 0 week). The dashed line separated preconception period, first trimester, second trimester and third trimester from left to right, respectively.


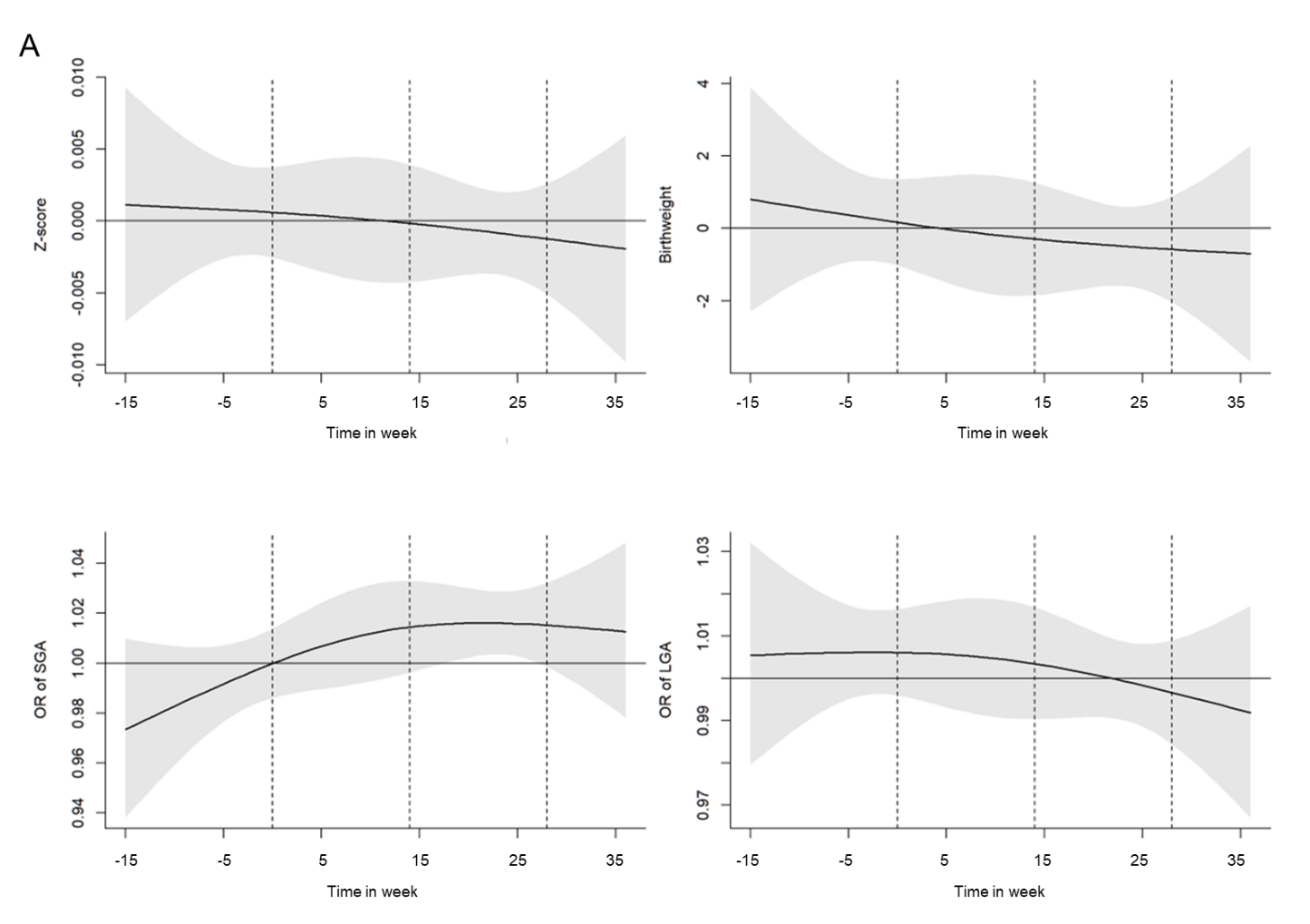


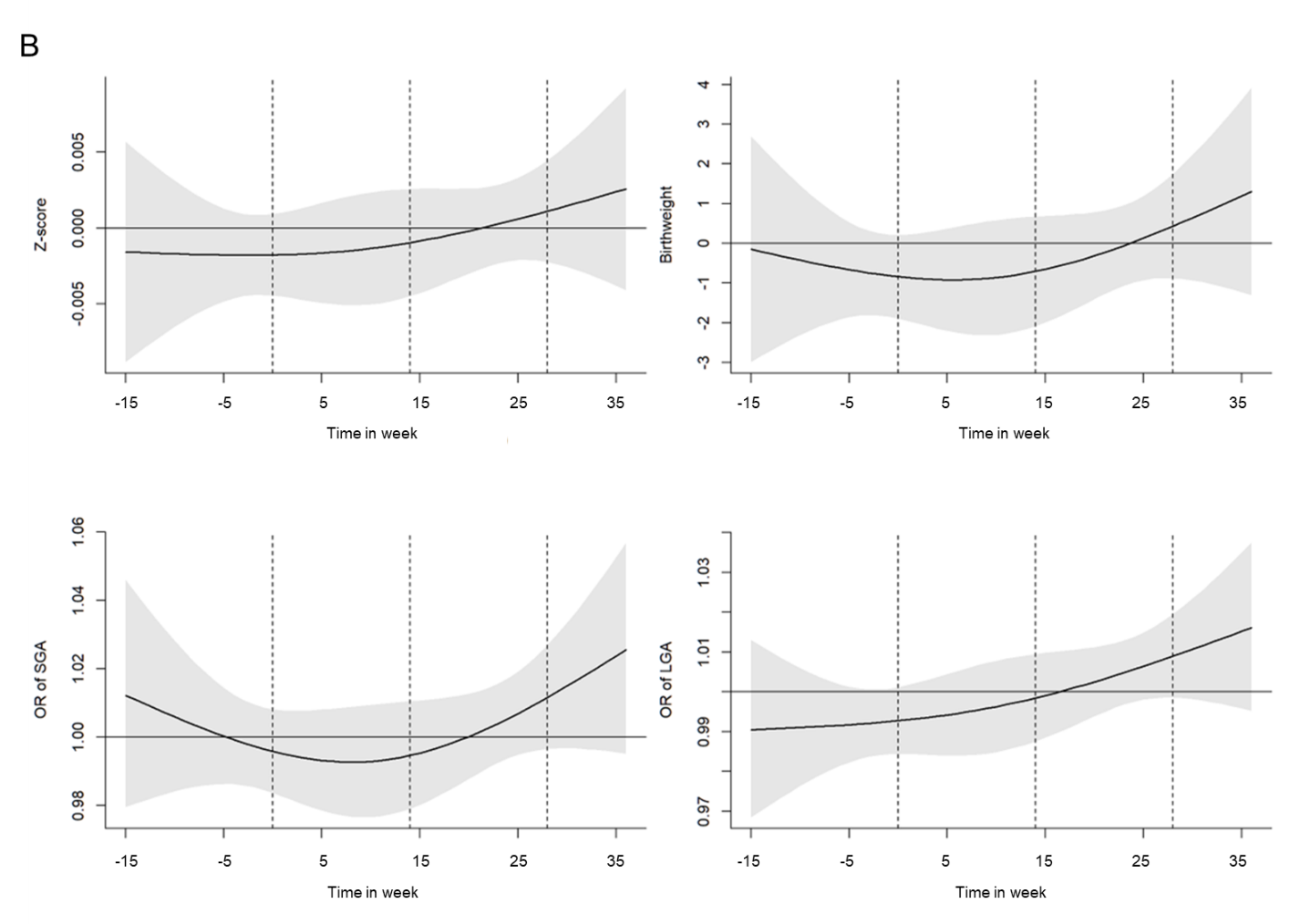


**Figure S5.** Plots of the differences in birthweight Z-score, birthweight, SGA and LGA associated with 1 IQR increase (16 µg/m^3^) of PM_2.5_ concentrations during preconception and gestational periods stratified by children’s sex (A: female, B: male). The x-axis indicated the time in week of gestation (i.e. conception date is 0 week). The dashed line indicated preconception period, first trimester, second trimester and third trimester from left to right, respectively.


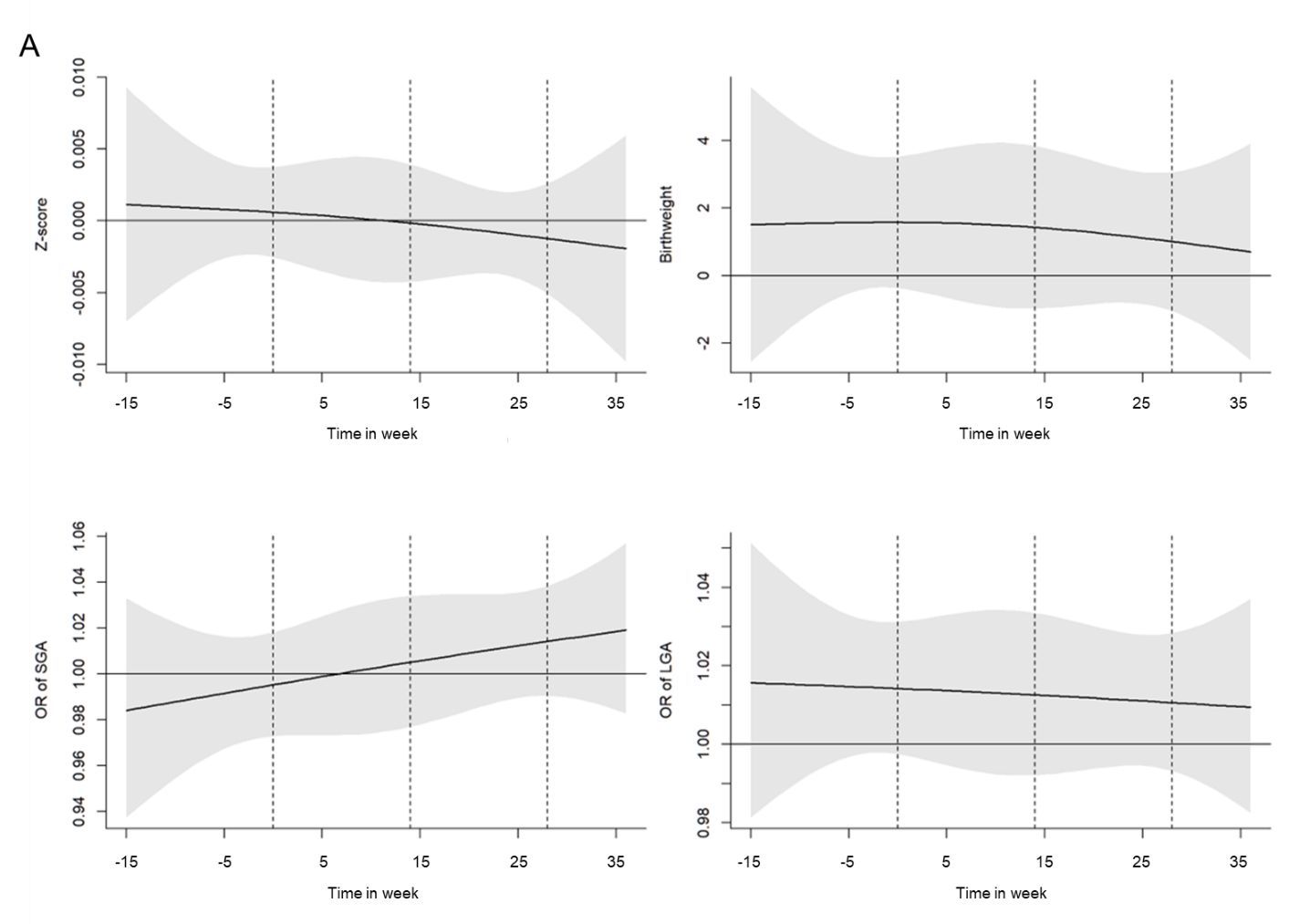


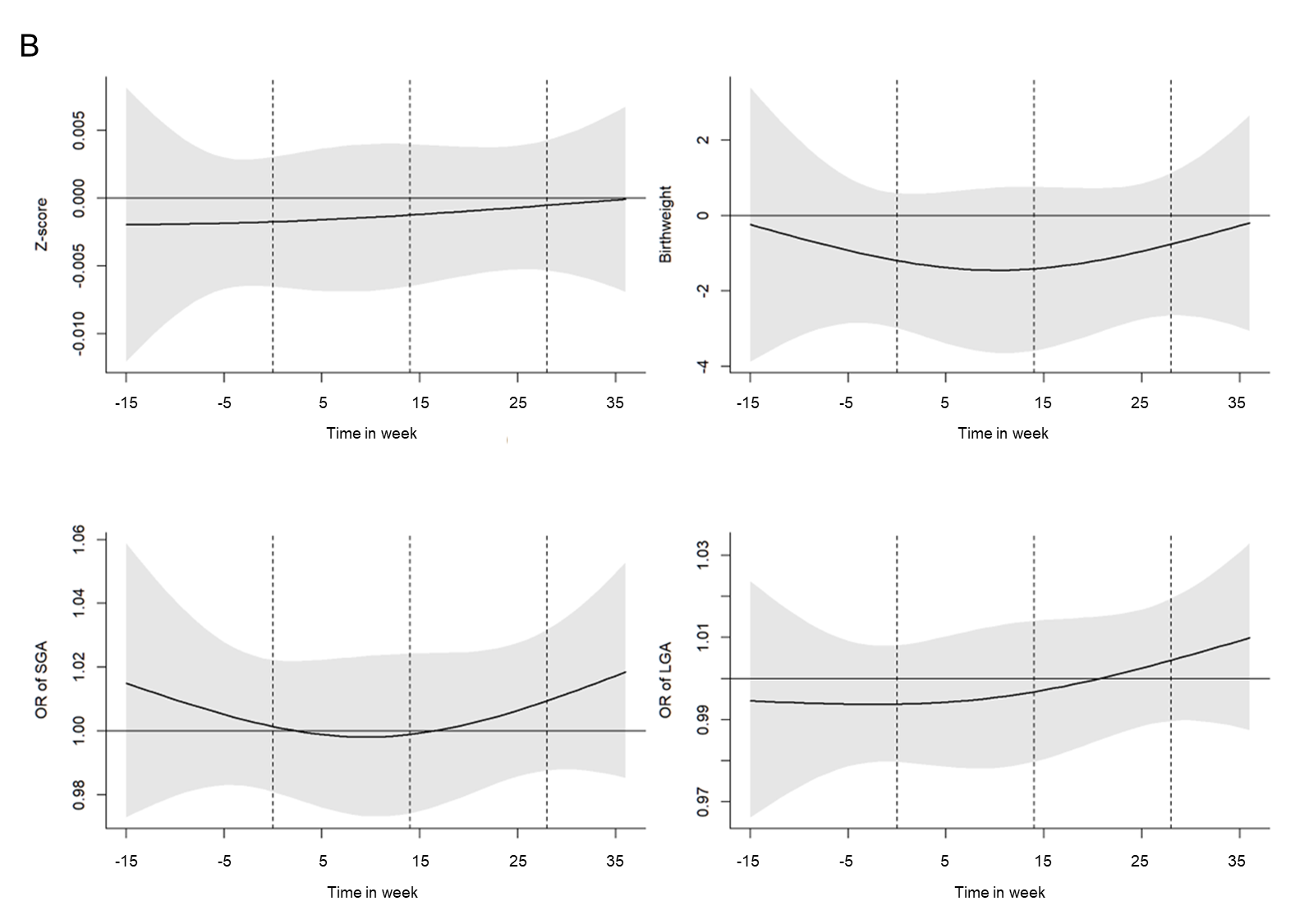


**Figure S6.** Differences in birthweight Z-score, birthweight, SGA and LGA associated with 1 standard deviation (SD) increase of (A) NO_2_ and (B) PM_2.5_ air pollution. Model adjusted for the maternal age, ethnicity, education, occupation, maternal BMI at first trimester, maternal gravidity, maternal smoking status, season of delivery, gestational age (only for birthweight), children’s sex (only for birthweight), temperature, and relative humidity.


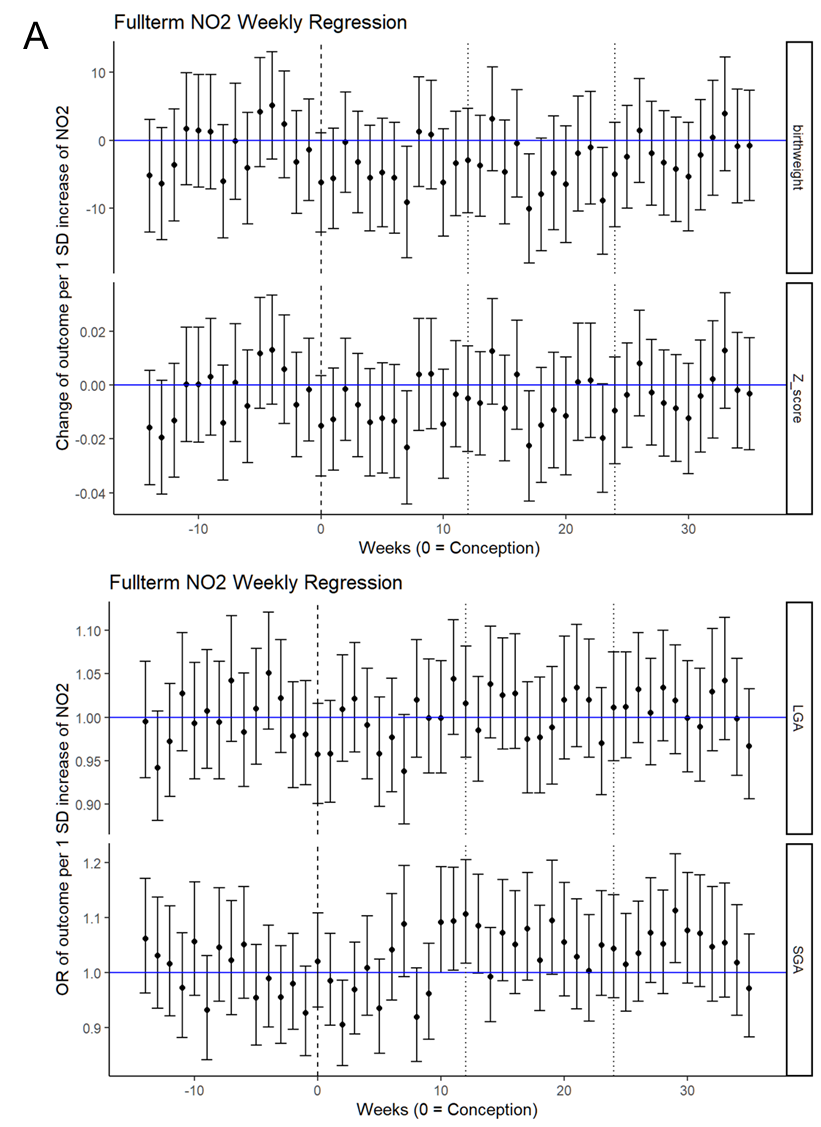


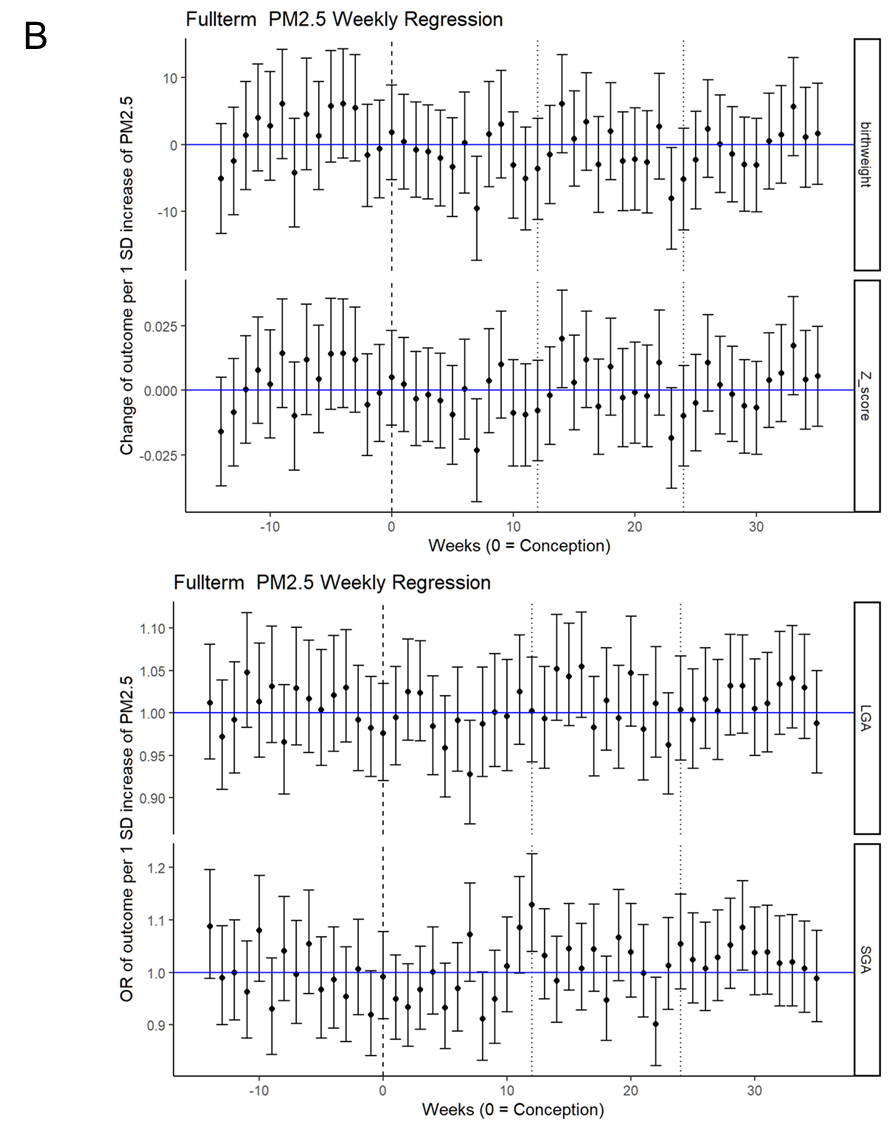

Supplement: Supplementary file 1 — Supplementary Material 1 [file 12940_2023_1022_MOESM1_ESM.docx]
